# Bald sea urchin disease shifts the surface microbiome on purple sea urchins in an aquarium

**DOI:** 10.1093/femspd/ftad025

**Published:** 2023-09-15

**Authors:** Chloe G Shaw, Christina Pavloudi, Megan A Barela Hudgell, Ryley S Crow, Jimmy H Saw, R Alexander Pyron, L Courtney Smith

**Affiliations:** Department of Biological Sciences, Suite 6000 Science and Engineering Hall, 800 22nd St NW, Washington DC 20052, United States; Department of Biological Sciences, Suite 6000 Science and Engineering Hall, 800 22nd St NW, Washington DC 20052, United States; Department of Biological Sciences, Suite 6000 Science and Engineering Hall, 800 22nd St NW, Washington DC 20052, United States; Department of Biological Sciences, Suite 6000 Science and Engineering Hall, 800 22nd St NW, Washington DC 20052, United States; Department of Biological Sciences, Suite 6000 Science and Engineering Hall, 800 22nd St NW, Washington DC 20052, United States; Department of Biological Sciences, Suite 6000 Science and Engineering Hall, 800 22nd St NW, Washington DC 20052, United States; Department of Biological Sciences, Suite 6000 Science and Engineering Hall, 800 22nd St NW, Washington DC 20052, United States

**Keywords:** Strongylocentrotus purpuratus, echinoderm, 16S rRNA high throughput sequencing, bacterial infection, microbiome

## Abstract

Bald sea urchin disease (BSUD) is most likely a bacterial infection that occurs in a wide range of sea urchin species and causes the loss of surface appendages. The disease has a variety of additional symptoms, which may be the result of the many bacteria that are associated with BSUD. Previous studies have investigated causative agents of BSUD, however, there are few reports on the surface microbiome associated with the infection. Here, we report changes to the surface microbiome on purple sea urchins in a closed marine aquarium that contracted and then recovered from BSUD in addition to the microbiome of healthy sea urchins in a separate aquarium. 16S rRNA gene sequencing shows that microhabitats of different aquaria are characterized by different microbial compositions, and that diseased, recovered, and healthy sea urchins have distinct microbial compositions, which indicates that there is a correlation between microbial shifts and recovery from disease.

## Teaser

The surface microbiome undergoes major shifts as purple sea urchins recover from the bald sea urchin disease.

## Introduction

Bald sea urchin disease (BSUD) is a bacterial infection that impacts many species of sea urchins and was first described in the red sea urchin, *Mesocentrotus franciscanus* (Johnson 1971). Spine loss is the key characteristic of the disease, hence the general name of BSUD. Since the original characterization, there has been a wide range of descriptions of BSUD that has mostly been based on the presence of discrete lesions on the surface of sea urchins and the loss of appendages within those lesions that may include test erosion and body wall perforation (Table 1). However, other symptoms include general spine loss in the presence or absence of surface lesions and disruption of the peristomial membrane. Sea urchins are known to recover completely from BSUD or may be at risk of death depending on the severity of the infection (Lafferty et al. 2004). Declines in echinoid populations as a result of disease can have significant consequences in marine systems because they may result in phase shifts in ecosystems depending on the extent of the echinoid population reduction from a mass die-off and whether the infected species is a keystone member of an ecosystem (reviewed in Smith et al. 2022). For example, in the 1980s the near disappearance of a keystone herbivore, the long-spined black sea urchin, *Diadema antillarum*, due to disease of unknown etiology resulted in a massive ecological phase shift in the Caribbean Sea from coral cover to uncontrolled algal growth on the reefs (Sammarco 1980, Vega Thurber et al. 2012, Smith et al. 2022). A repeat mass mortality event of *D. antillarum* beginning in 2022 in the Caribbean Sea shows general spine loss prior to death and the pathogen has been identified as a scuticociliate (Hewson et al. 2023). Echinoid diseases are also associated with substandard aquaculture practices including suboptimal water temperature, eutrophication, poor food quality, and injuries from handling and sorting (Tajima et al. 1998, Brink et al. 2019, Chi et al. 2022). Sea urchin disease results in economic losses for mariculture facilities and financial impacts on the mariculture industry (Wang et al. 2013a). Consequently, the causes of diseases in echinoids and the possibilities of their prevention are important to understand for both marine ecosystem structure and for successful mariculture.

**Table 1.** Infections on the surface of sea urchins show a wide range of symptoms.

Disease	Symptoms	Reference
BSUD	Spine loss without lesions	This paper, Clemente et al. (2014), Brink et al. (2019)
Black mouth disease or no name given	Spine loss without lesions	Chi et al. (2022), Tajima et al. (1988)
	Blackish peristomial membrane	
BSUD	Lesions on the body surface showing green, pink, red, and purple discoloration	Lafferty et al. (2004), Girard et al. (2011), Brink et al. (2019), Grech et al. (2022), Federico et al. (2023)
	Appendage loss within lesions	
BSUD or Spotting disease	Lesions of various colors	Maes and Jangoux (1984a,b), Gilles and Pearse (1986), Roberts-Regan et al. (1988), Becker et al. (2008), Wang et al. (2020)
	Appendage loss within lesions	
	Test erosion and body wall perforation	
Lesion syndrome disease or no name given	Lesions of various colors	Wang et al. (2013a), Hira and Stensvåg (2022)
	Appendage loss within lesions	
	Test erosion and body wall perforation	
	Blackish peristomial membrane	
Red spotting disease or no name given	Lesions of various colors	Nagelkerken et al. (1999), Virwani et al. (2021)
	Appendage loss within lesions	
	Spine loss over some or all of the surface	
Red spotting disease	Lesions of various colors	Li et al. (2020)
	Appendage loss within lesions	
	Spine loss over some or all of the surface	
	Blackish peristomial membrane	

Many reports of BSUD outbreaks have focused on identifying the underlying causative agent and have indicated that the pathogen is unlikely to be fungal, blue–green algal, or viral, but is likely bacterial (Maes and Jangoux 1984a, Wang et al. 2013b, Bauer and Young 2000). A wide variety of bacteria are associated with BSUD, and many can reproduce the disease (Table 2). Given this variety, which has been correlated with a wide range of oceanic locations, and given the plethora of potentially pathogenic bacteria in marine systems, there may be a vast number of causative agents of BSUD. This variety may be the basis for the range of disease symptoms that are evident on the surface of sea urchins (Table 1).

**Table 2.** Bacteria associated with BSUD and related surface diseases.

Sea urchin host	Bacteria	Pathogenic	Reference
*Strongylocentrotus purpuratus*	*Vibrio anguillarum, Aeromonas salmonicida*	Yes	Gilles and Pearce (1986)
*Paleopneustes cristatus*	*Vibrio alginolyticus*	Yes	Bauer and Young (2000)
*Strongylocentrotus droebachiensis*	*Acinetobacter* sp., *Alcaligenes* sp.	ND^1^	Roberts-Regan et al. (1988)
*Strongylocentrotus droebachiensis*	*Vibrio echinoideorum*	Yes	Hira and Stensvåg (2022)
*Tripneustes gratilla*	*Exiguobacterium* sp., *Vibrio* spp.	Yes^2^	Becker et al. (2007)
*Tripneustes gratilla*	Microbiome dysbiosis	ND	Brink et al. (2019)
*Paracentrotus lividus*	*Vibrio splendidus, V. gigantis*	ND	Grech et al. (2022)
*Paracentrotus lividus*	Unknown	ND	Maes and Jangoux (1984a)
*Strongylocentrotus intermedius*	*Vibrio* spp.	ND	Takeuchi et al. (1999)
*Strongylocentrotus intermedius*	*Flexibacter*	Yes	Tajima et al. (1997)
*Diadema africanum*	*Vibrio alginolyticus*	Yes	Clemente et al. (2014)
*Strongylocentrotus intermedius*	Community	Yes	Wang et al. (2013a)
	*Vibrio* spp., *Pseudoalteromonas tetraodonis, Shewanella aquimarina*		
*Strongylocentrotus intermedius*	*Vibrio coralliilyticus*	Yes	Li et al. (2020)
*Meoma ventricosa*	*Pseudoalteromonas haloplanktis tetraodonis*	Yes^3^	Nakelkerken et al. (1999), Richie et al. (2000)
*Paracentrotus lividus*	*Vibrio* sp., *Tenacibaculum* sp.	No	Federico et al. (2023)

1 ND, not done. The pathogenicity of the bacteria was not tested.

2 Injuries from the ectoparasitic gastropod, *Vexilla vexillum*, results in lesions similar to spotting disease.

3 Bacterial virulence was tested by injection into and survival of *Lytechinus variegatus*.

Although the presence and characteristics of BSUD for sea urchins in the ocean or in aquaculture facilities have been reported (Table 1) including the associated bacterial pathogens (Table 2), there are no studies that describe the onset and recovery of BSUD in closed aquaria in the absence of experimentally induced infection. Here, we report on purple sea urchins, *Strongylocentrotus purpuratus*, that were shipped from Southern California to Washington DC, and subsequently acquired BSUD after being housed in a recirculating marine aquarium. Symptoms included the loss of all primary spines, no discrete lesions as described in previous reports (Clemente et al. 2014, Brink et al. 2019), and the animals recovered from the disease, regrew their primary spines, and returned to what appeared to be a healthy state. Because housing was in a closed aquarium system, this controlled environment provided an opportunity to investigate the changes in the surface microbiome community associated with BSUD. Samples were collected from sea urchin surfaces during the disease, after recovery, and from healthy animals housed in a separate aquarium, plus aquarium seawater and used for sequencing the 16S rRNA gene amplicons. Results indicated that the surface microbiomes on the three groups of sea urchins had significantly different compositions suggesting a microbial shift during disease recovery, and a difference in the microbial community on sea urchins from different shipments housed in two different aquaria. We speculate that the changes in the local environment of the ocean vs. a closed aquarium system, and perhaps our standard antibiotic treatments undertaken for all newly shipped sea urchins, may have contributed to an altered community state of the microbiomes. The microbial shifts may have led to the onset of disease in the sea urchins with subsequent shifts associated with recovery.

## Methods

### Sea urchin husbandry

Purple sea urchins, *S. purpuratus*, were collected from the near-shore water in San Diego CA, transferred to the Southern California Sea Urchin Company (Corona del Mar, CA) and placed in holding tanks for 2 weeks in the open seawater system at the Kerckhoff Marine Laboratory (California Institute of Technology). Apparently healthy sea urchins (*n* = 40) that survived collection were purchased and shipped overnight to George Washington University in Washington DC. Sea urchins were housed in aquarium B (125 gallon) with recirculating ASW (Premium Marine Salt, OmegaSea), salinity of 32–35 ppt, 13–14°C, and outfitted with both physical and biofilters, a UV light housing, and a protein skimmer. The central aquarium pump (Pond-Mag 9.5, Pondmaster) that was positioned in the aquarium sump, circulated 950 gallons/hour through the system. The water quality was maintained with weekly seawater changes of 5 gallons that also served in solid waste removal.

Aquarium A (125 gallons) in our laboratory, held 40 healthy sea urchins that had been housed in that aquarium for 9 months, and for which no diseases had been observed. The salinity, temperature, filtering, and UV light housing were the same as for aquarium B, and the central pump circulated 1057 gallons/hour through the system (Marine DC pump DCT-4000, Jecod). All animals were fed weekly with rehydrated brown seaweed, *Saccharina angustata* (Kjellman) (WEL-PAC).

### Treatment of sea urchins with penicillin and streptomycin

Based on our standard protocol, upon arrival all shipments of sea urchins were treated by immersion for 1–2 hours at 14°C in a tray (8 l) of freshly prepared ASW with 12 mg/l penicillin and 50 mg/l streptomycin sulfate (pen/strep). After treatment, different sea urchin shipments were placed in different aquaria.

### Sample collection from sea urchin surfaces and from aquarium seawater

Samples were collected from randomly selected healthy sea urchins (*n* = 4, H1–H4) in aquarium A and diseased sea urchins (*n* = 4, D1–D4) in aquarium B, plus seawater samples from aquarium A (*n* = 2, WH1 and WH2) and aquarium B (*n* = 2, WD1 and WD2). Subsequent samples were collected from randomly selected recovered sea urchins (*n* = 4, R1–R4) in aquarium B as well as seawater samples (*n* = 2, WR1 and WR2). The cellular material collected from the surface of sea urchins was retained on nylon filters (0.22 μm, 47 mm diameter; GVS Filter Technology). Filters were held on a filter-holder assembly composed of a 300-ml funnel (Fisherbrand) that was spring clamped (Millipore, item XX1004703) to a fritted glass support base (Millipore) with a rubber stopper to insert into the top of a 1-l sidearm flask connected to the building vacuum system. Each sea urchin was placed in the funnel or was held over the vacuum filtration apparatus and 500 ml of seawater from its respective aquarium was poured slowly over all surfaces of the sea urchin, such that the material washed from the animal plus cells in the seawater were collected on the filter. The filters were inserted into individual 50 ml falcon tubes and stored at −80°C for later processing. After each collection, the filter apparatus and the funnel were rinsed with deionized water before collecting the sample from the next sea urchin. Seawater (500 ml) from each aquarium was filtered similarly to collect samples that served as controls. A sample of 500 ml of freshly mixed Omega ASW control was filtered in the same manner. Samples from diseased sea urchins were collected on day 140 after arrival and housing aquarium B at a time when all sea urchins were exhibiting the disease symptom of primary spine loss. Samples from recovered sea urchins were collected 300 days after arrival and housing in aquarium B, at a time when all sea urchins had regenerated their primary spines and were exhibiting normal behavior. Samples collected from healthy sea urchins in aquarium A were collected on the same day as when the diseased sea urchin samples were collected.

### Genomic DNA isolation from samples collected on filters

The genomic DNA (gDNA) isolation from nylon filters was carried out according to Turner et al. (2014) with modifications as tested with cultures of *E. coli* (Supplementary Data File 1). Each filter was placed in a sterile plastic petri dish (10 cm diameter), covered with 1 ml cetyltrimethylammonium bromide [CTAB; 2% CTAB, 100 mM Tris base (pH 7.4), 1.4 M NaCl, 1% polyvinylpyrrolidone, and 20 mM EDTA] and incubated for 10 minutes at 65°C with constant shaking. The filters were removed from heat and a cell scraper was used to remove all material from the filter, which was transferred to a 1.5-ml tube. Chloroform:isoamyl alcohol (24:1; 1 ml) was added to the CTAB solution, mixed by inversion, and centrifuged at 20,800 × *g* for 2 minutes at room temperature. The aqueous layer was transferred to a new tube, NaCl was added to 2.5 M, followed by the addition of an equal volume of 100% isopropanol and mixed by inversion. The samples were chilled at −80°C for 15 minutes and spun at 20,800 × *g* for 15 minutes at 4°C. The supernatant was discarded, then the pellet and the inside of the tube were washed with 150 μl of 70% ethanol, followed by a wash with 150 μl of 90% ethanol and air drying. gDNA pellets were resuspended in 10 μl Tris-EDTA buffer [TE; 10 mM Tris base (pH 7.4), 1 mM EDTA] and the concentration was evaluated on a spectrophotometer (NanoDrop 2000c, ThermoFisher). The gDNA size and level of degradation was evaluated with a 0.75% agarose gel with Tris-acetate-EDTA buffer (TAE; 40 mM Tris; 20 mM acetic acid, 1 mM EDTA) plus 1% ethidium bromide and imaged with a UV imaging system (Kodak Molecular Imaging, Kodak Gel Logic 1500 Imaging System).

### Polymerase chain reactions

The 16S rRNA gene was amplified from the isolated gDNA by PCR to test for the presence of prokaryotic DNA in the samples (27F, 5′AGA GTT TGA TCC TGG CTC AG; and 1492R, 5′ACG GTT ACC TTG TTA CGA CTT) that resulted in approximately a 1.5-kb amplicon (Weisburg et al. 1991). In total, two different concentrations of gDNA template (2 and 0.2 ng µl ^− 1^) were used in PCR (Bio-Rad T100 Thermal Cycler) and the final reaction volume of 20 µl contained 1X Primestar Buffer, 200 µM dNTPs, 0.3 µM each primer, and 0.5 units PrimeSTAR GXL DNA polymerase (Takara). The PCR program was 98°C for 1 minute, followed by 30 cycles of 98°C for 10 seconds, 60°C for 15 seconds, and 68°C for 15 seconds, with a final extension of 68°C for 1 minute and a 4°C hold. The amplicons were analyzed on a 0.8% agarose gel with TAE buffer plus ethidium bromide and imaged on an UV system as described above.

### 16S rRNA amplicon sequencing

The gDNA samples were processed and sequenced using the ZymoBIOMICS targeted sequencing service at ZymoResearch (Irvine CA). Targeted sequencing of the bacterial 16S rRNA gene was performed using the *Quick*-16S NGS Library Prep Kit (ZymoResearch) with custom-designed, proprietary primers to amplify the V3 and V4 region of the 16S rRNA gene. The library preparation was completed by quantitative real-time PCR (qPCR) and quantification of the PCR products using qPCR fluorescence readings. Library fragment sizes were selected and optimized with the Select-a-Size DNA Clean & Concentrator (ZymoResearch) and quantified with Tapestation (Agilent Technologies) and Qubit (ThermoFisher Scientific). The positive control sample used for library preparation was the ZymoBIOMICS Microbial Community DNA Standard (ZymoResearch). In addition to the ASW sample collected by DNA extraction from freshly made Omega seawater (see above), ZymoResearch employed a blank during library preparation, and both of these samples served as negative controls. The completed library was sequenced using a V3 reagent kit with 600 cycles on Illumina MiSeq, which was calibrated by a 10% spike-in of PhiX DNA. The raw sequence reads were uploaded to the Sequence Read Archive database at NCBI under the BioProject ID PRJNA851819.

### Amplicon sequence analysis

DADA2 (Callahan et al. 2016) was used to filter and trim sequences, infer amplicon sequence variants (ASVs) and remove sequencing errors and chimeric sequences. Taxonomy assignment was performed using SILVA release 138.1 (Quast et al. 2013). Phyloseq package (version 1.42.0) (McMurdie et al. 2013) was used to calculate alpha and beta diversity. Alpha diversity was estimated using the Observed Species, Chao1 (Chao 1984), and abundance-based coverage estimator (ACE) (Chao and Lee 1992) indices. Beta diversity was analyzed using Bray–Curtis distances (Bray and Curtis 1957) and visualized with nonmetric multidimensional scaling (nMDS).

Statistical significance of differences among groups for alpha diversity indices was performed using one-way ANOVA (*P* ≤ .05) and Tukey test in R. To evaluate statistical significance among groups for beta diversity, a permutational multivariate analysis of variance (PERMANOVA, *P* ≤ .05) using distance matrices was performed with the adonis2 function (Permutations = 999) with the vegan package (version 2.6.4) (Oksanen et al. 2015). Taxonomic groups that had significant differences in abundance among different groups were identified by Linear Discriminant Analysis Effect Size analysis (LEfSe) (Segata et al. 2011) using the microbiomeMarker package (version 1.3.2) (Cao 2022). The Upset plot was generated using UpSetR (version 1.4.0) (Gehlenborg 2019) and ComplexUpset (version 1.3.3) (Lex et al. 2014, Krassowski 2020). Rarefaction curves were generated using the MicrobiotaProcess package (version 1.6.6) (Xu and Yu 2022). The aforementioned analyses were performed using R version 4.1.1 (R Core Team 2021). The R code with complete pipeline utilized can be found in the GitHub repository https://github.com/chloeshaw8/Bald-sea-urchin-disease_project.

### Amplicon sequence analysis by Zymo Research

The sequenced reads were also evaluated by the 16S rRNA Amplicon Sequencing Data Interpretation service at ZymoResearch using their bioinformatics pipeline and analysis (Supplementary Data File 3). Briefly, the DADA2 pipeline was used to infer ASVs from raw reads, to remove errors and chimeric sequences, and taxonomic assignment was performed using the internal curated ZymoResearch dataset.

## Results

### Progression of BSUD shows a complete loss of primary spines followed by recovery and spine regrowth

**Figure 1.**
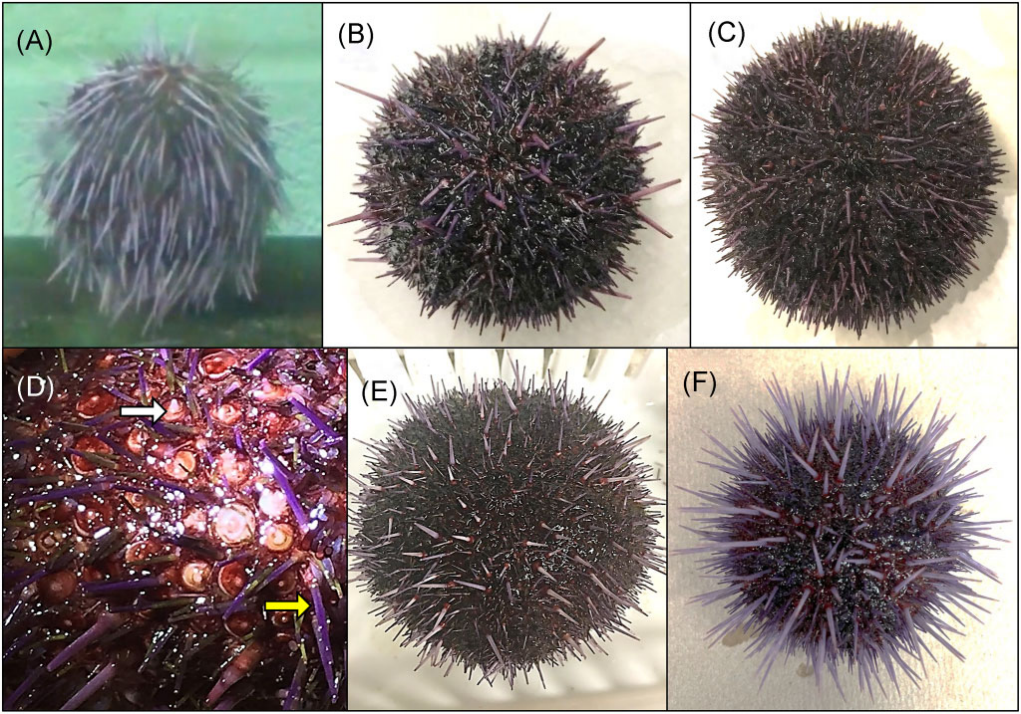
BSUD progression and recovery in the purple sea urchin. (A) An early symptom of BSUD is drooping spines suggesting that the sea urchin may be sleeping as has been observed on rare occasions for healthy sea urchins. Alternatively, the surface infection that would impact the muscles associated with the base of the spines and the tubercles may have altered spine movement (see Video 1; Supplementary Data File 2, Video 1 Legend). (B) A sea urchin infected with BSUD that has lost many of its primary spines. (C) A sea urchin with BSUD that has lost all of its primary spines and shows the red tubercles to which primary spines are normally attached. Shorter and smaller secondary spines remain attached. (D) A magnified image of the surface of a sea urchin with BSUD that has lost all primary spines. The secondary spines (yellow arrow), pedicellariae that are too small to see in this image, and tube feet remain on the animal surface. The beginnings of newly growing spines (white arrow) are present on some tubercles. (E) A recovering sea urchin with newly regrowing primary spines that are short, pointed, and light purple. (F) A healthy sea urchin after full recovery from BSUD with the characteristic of rigid primary spines oriented perpendicular to the animal body.

Sea urchins shipped across the country generally arrive stressed and spawning, and the survival of different shipments of sea urchins can range from as much as 80% to as poor as no survivors. Consequently, since 2012 we have employed a standard protocol for sea urchin care to improve survival. Sea urchins (*n* = 40 per shipment) received from California were treated upon arrival (day 0) by immersion in pen/strep for at least an hour at 15°C, which was adapted from standard culturing conditions for embryos and larvae (Leahy 1987, Adams et al. 2019, Schuh et al. 2020). Because the pen/strep treatment resulted in higher survival after shipping, it was incorporated for all sea urchins since implementing this treatment. Although the polychete, *Flabesymbios commensalis*, has been noted on sea urchins in some shipments (unpublished observations), none were evident on the animals in aquarium B, and no other ectoparasites were present (Becker et al. 2007, Virwani et al. 2021). On day 91 after arrival, multiple sea urchins in aquarium B showed unusual behavior of drooping spines (Fig. 1A), although when disturbed by sound or touch, they quickly reoriented their spines to the normal position of pointing directly out from the spheroid body (Video 1; Supplementary Data File 2, Video 1 Legend). On days 93–108, the sea urchins were constantly moving their spines, pointing them in different directions rather than the expected orientation of perpendicular from the body surface, which was another unusual behavior. By day 108, many sea urchins in aquarium B showed spine loss (Fig. 1B), and the animals with the greatest spine loss were placed in floating plastic boxes to minimize their interaction with other sea urchins. However, isolation in boxes did not reduce or block the spread of the disease to all animals in the aquarium. In an effort to save the animals from progression to death (which was assumed at the time), the weekly seawater change was increased from 5 to 10 gallons. Seawater chemistry for pH, ammonia, nitrate, nitrite, copper, and phosphorus for aquarium B and aquarium A, which housed healthy sea urchins, were normal and deemed not to be the basis for the diseased sea urchins in aquarium B. On day 113, a 20-gallon seawater change was carried out and the sea urchins were treated with a second immersion in pen/strep for 2 hours at 15°C. On day 116, the UV light bulbs for both aquaria A and B were changed to ensure that they were working optimally to sterilize the microbes in the seawater. By day 132, all sea urchins in aquarium B were diseased and had lost all primary spines, although the secondary spines (Fig. 1C and D, yellow arrow), pedicellariae, and tube feet remained intact. On day 132, sea urchins in aquarium B were treated a third time by pen/strep immersion, with the exception of nine animals that held tightly to the aquarium walls and could not be removed without significant injury. The sea urchins showing BSUD symptoms were difficult to grasp because their surfaces were abnormally slimy, which was consistent with infection and tissue disintegration (Fig. 1C and D). Throughout the infection, some sea urchins held tightly to the walls of the aquarium yet also showed the opposite behavior of failing to hold kelp securely during feeding. Notably, all animals with BSUD fed throughout the disease and none showed discrete lesions as described in other reports of spotting disease and some reports of BSUD (Table 1). From days 154 to 246, all sea urchins began to recover from the disease as evidenced by regrowing their primary spines (Fig. 1D, white arrow, and E) and returned to what appeared to be a healthy state (Fig. 1F).

### gDNA isolated from the surface of sea urchins includes bacterial DNA

Because the symptoms of BSUD were restricted to the external surface of the sea urchins, we reasoned that the surface microbiome was involved in disease onset. All sea urchins were fed the same diet, therefore, we also reasoned that the gut microbiome was not involved in BSUD. Furthermore, because a limited number of sea urchins can be delivered per shipment, we opted to save as many animals as possible and to investigate the surface microbes rather than sacrificing animals for tissue dissection. This also removed the problem of contamination of tissue samples with microbes released from the gut during dissection. When evaluating the gDNA (larger than 10 kb), more was isolated from the surface of the sea urchins than from the seawater samples (Supplementary Data File 2; Table S1, Supporting Information) although it was likely that the gDNA was a mixture from both sea urchin cells and surface fauna (Supplementary Data File 2; Figure S1A, Supporting Information). To test for the presence of bacterial DNA in the samples prior to sequencing, we evaluated the gDNA by PCR amplification of the 16S rRNA gene using our primers (see the section “Polymerase chain reactions”). Amplification of the 16S rRNA gene using the gDNA as the template (0.2 and 2.0 ng) resulted in the expected amplicons of 1.5 kb (Supplementary Data File 2; Figure S1B–E, Supporting Information). This indicated that bacterial gDNA was present in all samples in sufficient quantities to support subsequent microbiome analysis.

### Sufficient sampling depth is reached and unique ASVs are identified

Sequencing for all gDNA samples resulted in a total of 283,297 raw sequence reads following filtering and processing (Supplementary Data File 2; Table S2, Supporting Information). A total of 41,217 ASVs were inferred and after taxonomic assignment, 31 phyla were identified (Supplementary Data File 2; Table S3, Supporting Information). Rarefaction curves reached plateaus indicating sufficient sampling depth had been achieved for all samples (Supplementary Data File 2; Figure S2, Supporting Information). The sequenced reads were also analyzed by the ZymoResearch 16S Amplicon Sequencing Data Interpretation service using their own bioinformatics pipeline and analysis (Supplementary Data File 3). We chose to compare the ZymoResearch pipeline results, which utilizes their own internal database, to our results. The rarefaction curves for the two pipelines were similar (Supplementary Data File 2; Figure S2, Supporting Information; Supplementary Data File 3; Figure S1 and Table S1, Supporting Information), however, the filtering and processing of the reads resulted in the identification of 25 phyla for the ZymoResearch pipeline (Supplementary Data File 3; Table S2, Supporting Information).

### The microbiomes identified for each group are distinct

#### Three distinct microbiome compositions were identified for the three groups of sea urchins

**Figure 2.**
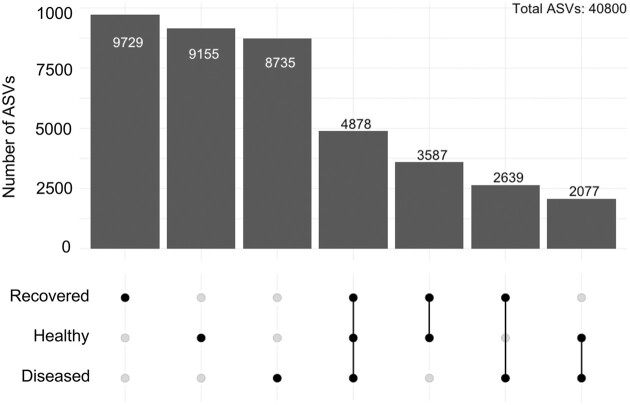
There are many ASVs identified in the microbiomes for each group of sea urchins. Upset plot shows that the microbiomes for the diseased, recovered, and healthy groups have many ASVs that are unique. Each bar shows the ASV count and the group in which the ASVs were identified is denoted directly below each bar. Shared ASVs are indicated by the line connecting groups below the bar indicating number of ASVs counted. Of the total number of ASVs ( 40,800), 4,878 (12%) ASVs are shared among the three groups, and the diseased and healthy groups have the fewest number of shared ASVs (5.1%).

**Figure 3.**
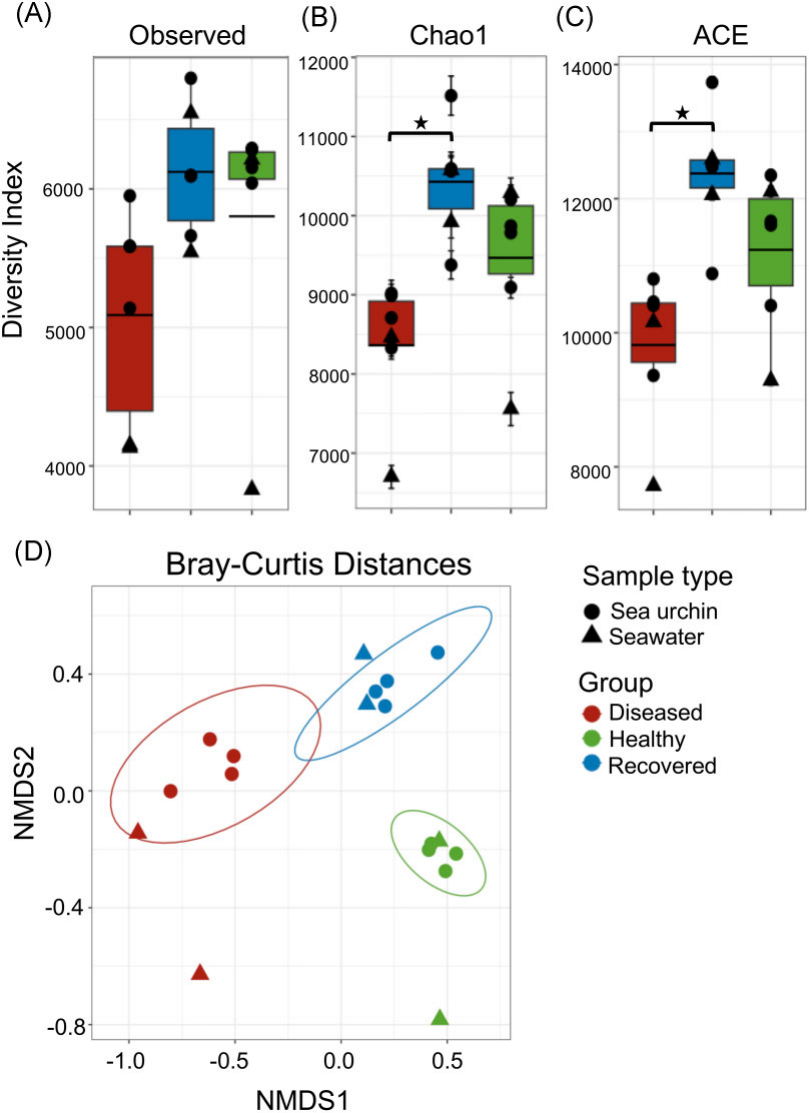
The surface microbiomes of sea urchins with BSUD have decreased alpha diversity compared to both recovered and healthy sea urchins. Alpha diversity was analyzed by (A) the Observed Species index, (B) the Chao1 index, and (C) the ACE index. Groups include microbiomes collected from the sea urchins and microbes in the aquarium seawater. The box plots show the average and quartile values for each group. The Chao1 index and the ACE index show significant differences between the diseased and recovered groups (ANOVA, *P* < .05). Error bars in the Chao1 index indicate the result as an estimate of diversity. (D) The microbial compositions of the surface microbiome are different among the three groups of sea urchins. Bray–Curtis distances estimates of beta diversity shows distinct differences for the bacterial composition and membership of ASVs in the microbiomes among the three groups of sea urchins. Ellipses show the 95% confidence intervals for the samples collected from sea urchins in each group.

The surface microbiomes sampled for the diseased, recovered, and healthy sea urchin groups each had many unique ASVs (Fig. 2) even though there were nearly 5000 shared ASVs. The recovered and healthy groups shared the most ASVs, whereas the diseased and healthy groups had the fewest number of shared ASVs. The diversity of the microbiomes was evaluated for alpha and beta diversity using several metrics (Fig. 3). The surface microbiome samples of the diseased sea urchins had decreased alpha diversity compared to the surface microbiome samples from the healthy and recovered sea urchins based on results from all metrics (Fig. 3A–C). Analysis by Chao1 and ACE identified significant differences in the alpha diversity of the surface microbiomes for the diseased compared to the recovered groups, but not between the diseased and healthy groups (ANOVA, *P* < .05). The microbiome samples from the recovered group had the greatest alpha diversity based on all indices, however, the samples showed no significant differences compared to the microbiome samples from healthy group. Although the ZymoResearch pipeline did not identify any significant differences in the alpha diversity among the groups, results were similar to our analysis. The Chao1 and Shannon metrics showed that the microbiomes on the sea urchins in the diseased group had lower alpha diversity based on compared to the recovered and healthy groups (Supplementary Data File 3; Figure S2, Supporting Information). Beta diversity based on Bray–Curtis distances revealed clustering of the samples from within each group and showed minimal overlap of clusters (Fig. 3D). This indicated that each microbiome sample within a group had a similar microbial composition and that the different groups (diseased, recovered, and healthy) had significantly different microbial compositions (PERMANOVA, *P* < .05). A nearly identical result was obtained from the ZymoResearch pipeline that showed significantly different microbial compositions among the groups (Supplementary Data File 3; Figure S3, Supporting Information).

#### Many phyla show shifts in abundance as the sea urchins recover from BSUD

**Figure 4.**
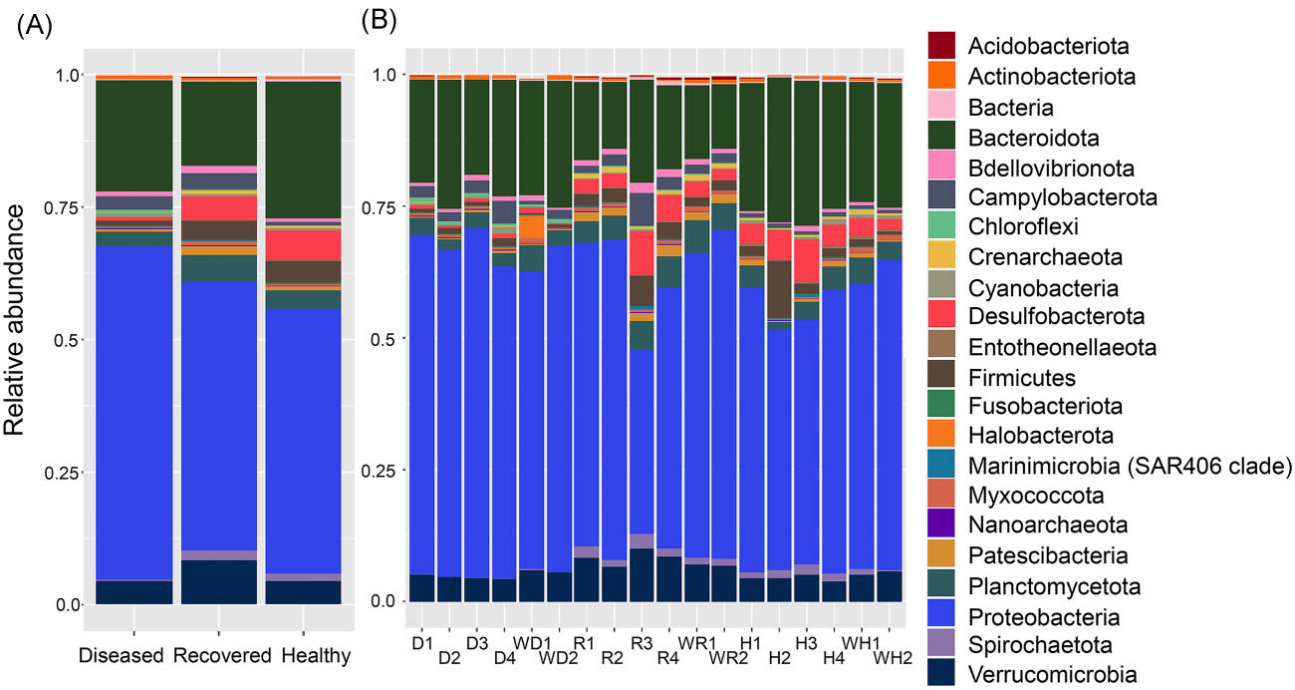
The surface microbiomes are dominated by Proteobacteria and Bacteroidota. The phyla shown have an average relative abundance of > 0.1% for at least one sample across the groups (Supplementary Data File 2; Table S3, Supporting Information). The taxa are shown as (A) the average relative abundance in the microbiomes for each group, and (B) the relative abundance for samples collected from each sea urchin in the three groups as well as the water samples. Samples from sea urchins are indicated in (B) as diseased (D), recovered (R), and healthy (H), and the numbers (1–4) correlate with the four sea urchins within each group. Water samples are indicated with a (W).

To evaluate the taxa in the microbiomes from the three groups of sea urchins, the phyla with an average relative abundance of > 0.1% across all samples were selected for comparisons among the three groups (Supplementary Data File 2; Tables S3 and S4, Supporting Information). Results showed that all microbiomes were similarly dominated by Proteobacteria and Bacteroidota (Fig. 4). The most abundant phyla showed minor differences among the three groups (Fig. 4A), and these differences were evident for each of the samples from the sea urchins within each group (Fig. 4B). A few phyla had different abundances among the groups, such as Desulfobacterota, Spirochaetota, and Firmicutes that showed reduced abundances in the microbiome samples of the diseased group compared to samples from the recovered and healthy group microbiomes. The ZymoResearch pipeline similarly showed that all microbiomes were dominated by Proteobacteria and Bacteroidota, and that there were a few phyla that differed in abundance among the groups, including Spirochaetota, Firmicutes, and Verrucomicrobia (Supplementary Data File 3; Figure S4 and Table S2, Supporting Information). Because these changes were evident at the level of phylum, this indicated significant changes to the microbiomes. Furthermore, differences in the abundances of certain phyla suggested that the microbiomes on the diseased sea urchins were altered and that changes to the microbiomes occurred as the sea urchins recovered from the BSUD infection.

#### The microbiomes on the three groups of sea urchins show differences in microbial composition

**Figure 5.**
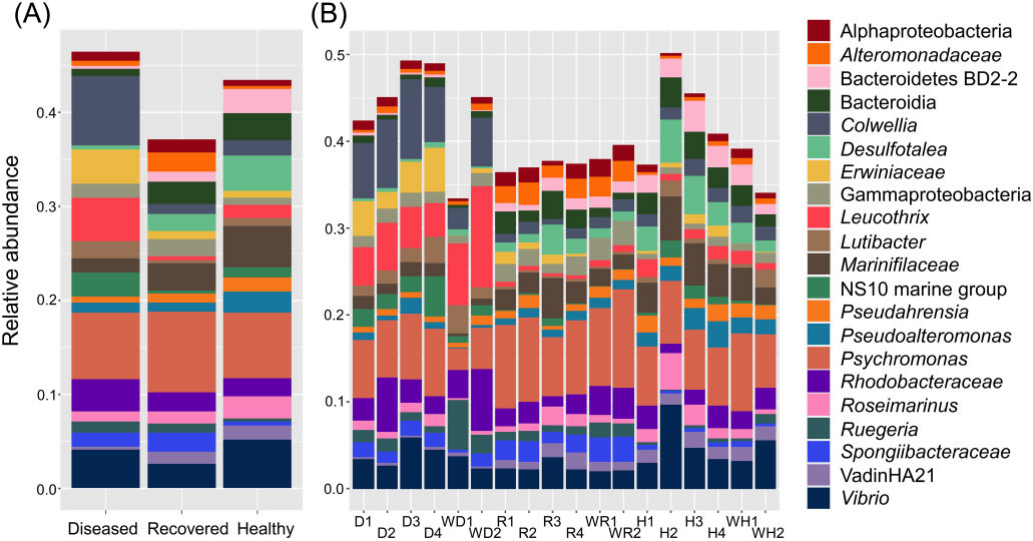
The most abundant genera in the microbiomes differ among the three groups of sea urchins. The genera shown have an average relative abundance of > 1% for at least one sample (Supplementary Data File 2; Table S5, Supporting Information). The results show (A) the average relative abundance of microbial taxa from all sea urchins in each sample group and (B) the relative abundance of taxa for samples collected from each sea urchin in each of the three sample groups as well as the water samples. Taxa are listed to the right, and ASVs that could not be assigned a genus are indicated as the next matching taxonomic level. Samples from sea urchins are indicated in (B) as diseased (D), recovered (R), and healthy (H), and the numbers (1–4) correlate with the four sea urchins within each group. Water samples are indicated with a (W).

Taxa identified in the microbiomes were also evaluated at the genus level and those with an average relative abundance of > 1% for at least one sample across all groups were selected and evaluated for their relative abundance per sample (Fig. 5; Supplementary Data File 2; Table S5, Supporting Information), which included the seawater samples for comparison (Supplementary Data File 2; Table S6, Supporting Information). Major differences in the compositions of the microbial genera were evident among the three groups (Fig. 5A) and differences were also evident for all samples collected within the groups of sea urchins (Fig. 5B). *Psychromonas* and *Vibrio* had similar abundances in all three groups, however, the majority of the most abundant genera had abundances that differed among the three groups (see the GitHub repository for the complete table). For example, the genera *Colwellia, Leucothrix*, and a genus from the family *Erwiniaceae* were highly abundant in the diseased group microbiomes compared to the recovered and healthy group microbiomes. Alternatively, *Desulfotalea* and a genus of the family *Marinifilaceae* were lower in abundance in the diseased group microbiome compared to the recovered and healthy group microbiomes. Similarly, the ZymoResearch pipeline showed that *Psychromonas* and *Vibrio* had similar abundances among all three groups, but that many other genera differed in abundance, such as *Colwellia, Leucothrix*, and *Erwinia*, which were elevated in the diseased group microbiomes (Supplementary Data File 3; Figure S5 and Table S3, Supporting Information). These results suggested distinct microbial compositions of the microbiomes for the three groups of sea urchins, which was in agreement with significant differences identified for beta diversity (Fig. 3D).

#### Major shifts to the microbial composition are associated with recovery from BSUD

Because the microbiome samples collected for the diseased and recovered groups of sea urchins were obtained at different times from the same animals housed in aquarium B, changes in the taxa on their surface microbiomes could be compared directly to identify taxa associated with BSUD. Notably, the surface microbiomes of the diseased group had elevated abundances of *Colwellia, Erwiniaceae, Leucothrix, Lutibacter*, and NS10 marine group, compared to the microbiomes on the sea urchins after recovery (Fig. 5). Alternatively, the recovered group had elevated abundances of *Alteromonadaceae*, Bacteroida, *Desulfotalea*, Gammaproteobacteria, and *Marinifilaceae* compared to the diseased group samples. The ZymoResearch analysis identified many similar genera as elevated in the microbiomes of diseased sea urchins, including *Erwinia, Leucothrix, Cowellia*, and *Lutibacter*. Furthermore, many similar taxa were also identified as elevated in the recovered group including *Desulfotalea* and *Alteromonas* (Supplementary Data File 3; Figures S5, S6, and Table S3, Supporting Information). In contrast to our pipeline, the ZymoResearch pipeline assigned matches to species for many taxa, which identified species within genera that were either elevated or reduced in the diseased group microbiomes (Supplementary Data File 3; Figures S7 and S8, Supporting Information). For example, species *Colwellia meonggei, Leucothrix mucor*, and *Erwinia rhapontici* were elevated in the diseased group microbiomes (Supplementary Data File 3; Figure S7A–C, Supporting Information). These taxonomic differences suggested that as the sea urchins recovered from BSUD, the species membership on their surface microbiomes underwent major shifts in composition.

#### Different aquaria show distinct microbial compositions

Microbiome samples collected from recovered and healthy sea urchins were both from noninfected sea urchins but were housed in different aquaria. Therefore, a comparison among the microbial compositions for these two groups were used to identify differences attributed to different shipments of sea urchins and to housing in separate aquarium environments. The genera in the microbiomes of the recovered sea urchins in aquarium B had elevated *Alteromonadaceae*, Gammaproteobacteria, *Ruegeria*, and *Spongiibacteraceae* compared to the microbiomes of the healthy sea urchins in aquarium A (Fig. 5). In contrast, the healthy sea urchin microbiomes had elevated Bacteroidetes BD2-2, *Desulfotalea, Leucothrix, Marinifilaceae*, and *Roseimarinus* compared to the microbiomes from the recovered group. Results from the ZymoResearch pipeline identified many taxonomic differences between the recovered and healthy group microbiomes, but the results differed from the taxa identified by our pipeline (Supplementary Data File 3; Figures S5, S6, and Table S3, Supporting Information). For example, taxa elevated in the recovered group microbiomes were *Alteromonas, Sulfurimonas*, and *Cobetia*, and taxa elevated in the healthy group microbiomes were *Parvularcula, Pseudoalteromonas*, and *Neiella*(Supplementary Data File 3; Figure S6, Supporting Information). Nonetheless, both evaluations of the taxonomic differences indicated distinct microbial compositions of sea urchins from different shipments housed in different aquaria.

#### The major differences in microbial composition based on the most abundant taxa are demonstrated for the three groups of sea urchins

**Figure 6.**
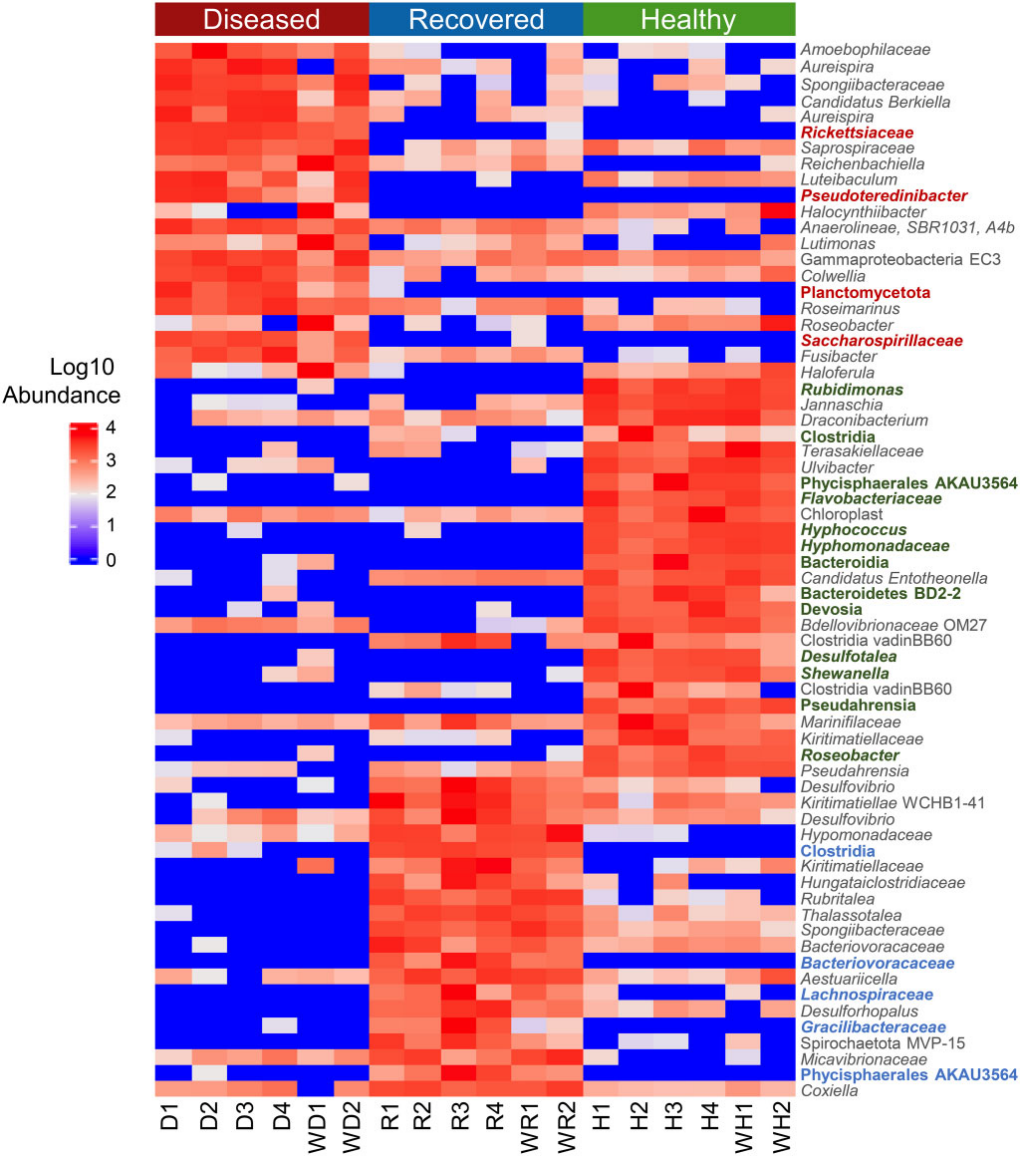
Many microbial taxa are significantly differentially abundant among the groups. A heatmap shows the relative abundance of the taxa in each sample within the three groups including the water samples. Identified taxa are ASVs and the lowest known taxonomic classification is listed. Taxonomic names that are colored have a notably reduced abundance or are completely absent in the other two group microbiomes. The taxa displayed have an LDA score of > 3.1 based on identification by LEfSe. All taxa with an LDA score of > 3.0 along with their LDA score and *P*-values are available in Supplementary Data File 2; Table S7 (Supporting Information). The threshold was increased for better visualization of the data.

In addition to evaluating the most abundant taxa, those that showed significant differences among samples were evaluated using LEfSe (Supplementary Data File 2; Table S7, Supporting Information). The most abundant taxa in the microbiomes from the three groups of sea urchins were generally different (Fig. 6). The differentially abundant taxa in the microbiomes of the diseased group relative to the other two groups were also some of the most abundant taxa. Therefore, these taxa may be key to the composition of the microbiomes on sea urchins with BSUD and included ASVs of the genera *Colwellia, Leucothrix, Roseimarinus*, and the families *Spongiibactereaceae* and *Rhodobacteraceae*. There were also differentially abundant taxa that were not the taxa of highest abundance, which may still be key to the composition of the microbiomes. Notably, families from which multiple ASVs were identified as differentially abundant in the diseased group microbiomes included *Saprospiraceae*, of which two ASVs were within the genus *Aureispira*. Furthermore, five ASVs were identified in the family *Roseobacteraceae*, of which two were within the genus *Halocynthiibacter*, and there were three ASVs in the family *Flavobacteriaceae*. Results from LEfSe in the ZymoResearch pipeline identified many similar differentially abundant taxa in the diseased group microbiomes, including *C. meonggei, Lutibacter agarilyticus, L. mucor*, and a genus of the family *Saprospiraceae*, among others (Supplementary Data File 3; Tables S4 and S5, Supporting Information). Many taxa were also differentially abundant in the recovered and healthy group microbiomes (Fig. 6). Notably, the recovered group microbiomes had multiple ASVs of the genera *Clostridia* and *Desulfovibrio*, whereas ASVs of the genus *Desulfotalea, Marinifilaceae*, and *Pseudahrensia* were differentially abundant in the microbiomes of the healthy group. The results of the LEfSe analysis in the ZymoResearch pipeline overlapped with the results of our pipeline and included *Desulfovibrio* and *Coxiella* as differentially abundant in the recovered group microbiomes, and *Desulfotalea, Shewanella*, and *Draconicbacterium* in the healthy group microbiomes (Supplementary Data File 3; Tables S4 and S5, Supporting Information). These differentially abundant taxa identified by LEfSe for both pipelines in the microbiomes for the recovered and healthy groups defined the differences between the two shipments of sea urchins that were housed in different aquaria. The differences among the microbiomes of the three groups were in agreement with the beta diversity results (Fig. 3) and identified the set of key taxa underlying the differences in microbial composition. Overall, differential abundances of taxa showed that as sea urchins transitioned from infection to recovery, their microbiomes underwent major shifts in bacterial composition. This was in agreement with the evaluation of taxa that were of the greatest abundance. Furthermore, differential abundances were also evident for sea urchins from different shipments housed in different aquaria.

## Discussion

### The characteristics of BSUD are highly variable

The BSUD outbreak in aquarium B showed consistent symptoms for every sea urchin, including unusual primary spine movement and positioning before their loss over the entire surface of the animals, in addition to abnormal tube foot behavior and damage to the epidermal tissue. Disease recovery was evident based on regrowth of the primary spines for all animals. Because the symptoms were observed more than 90 days after the arrival of the sea urchins, the basis for the disease onset is not understood but may have been a combination of several possible factors. At least some of the microbes that arrived with the sea urchins housed in aquarium B may have been involved in the disease onset, in addition to unknown effects of the pen/strep treatments on the microbiomes, a shift in the surface microbiome that occurs when sea urchins are moved from the ocean to a closed aquarium (Wessel et al. 2022), and perhaps the microbial environment of aquarium B in which they were housed. The recovery from BSUD infers that the skeletogenic cells, which are involved in the regeneration process, are either not affected by BSUD, or that these cells migrate from noninfected regions of the body including the subdermal test onto the spine tubercles to function in rebuilding the primary spines (Heatfield 1970, 1971, Heatfield and Travis 1975, Märkel and Röser 1983, Dubois and Ameye 2001, Politi et al. 2004, Reinardy et al. 2015, Emerson et al. 2017). BSUD symptoms reported here differ somewhat from most other reports of BSUD, spotting disease, and other similar diseases in echinoids (Table 1). The majority of reports describe spine loss from discrete lesions as the most common characteristic of the disease (e.g. Maes and Jangoux 1984b), however, there are a few reports of BSUD showing general spine loss in the absence of lesions (Clemente et al. 2014, Brink et al. 2019, this paper). The wide range of BSUD symptoms suggests significant variation in the pathogenicity of the infection, which infers that many different microbes and/or opportunistic bacteria may underlie this variable pathology and disease severity. The microbial composition of BSUD reported here may not have been as pathogenic as in other reports given that the sea urchins recovered from the disease in our facility, whereas BSUD has been documented to cause mass mortalities in marine environments (Pearse et al. 1977, Boudouresque et al. 1980, Azzolina et al. 1985). Variation in the bacterial composition of diseased sea urchins based on sequence data is not unusual and has been reported previously in which sea urchins from different locations in the ocean show distinct bacterial compositions associated with discrete lesions (Becker et al. 2008). This may be a basis for the differences in the microbiomes reported here for the two shipments of animals housed in different aquaria. This concept is further supported by the taxa identified in the diseased microbiome for the sea urchins in aquarium B, which differ greatly from the taxa identified in other reports of BSUD (Gilles and Pearce 1986, Becker et al. 2008, Brink et al. 2019). Our results suggest that the bacterial composition associated with BSUD can include a variety of bacterial species that can differ based on the location of the sea urchins, suggesting that many combinations of bacteria may cause symptoms consistent with BSUD. Overall, the variation in the symptoms described in reports of BSUD suggest that differences in the microbiomes and/or the types of opportunistic bacteria may impact the level of symptoms and severity of the disease. Based on results presented here, we suggest that the observed symptoms, including spine loss and surface discoloration are general indicators of many different sea urchin diseases that fall within the category of BSUD.

### Many factors may contribute to an altered microbial community

Our findings for *S. purpuratus* indicate that the microbiome on diseased sea urchins is distinct from that on nondiseased sea urchins, and that recovery from disease is associated with significant changes in the bacterial composition of the surface microbiome. Furthermore, the similarities between the microbiome compositions on the sea urchins compared to that in the aquarium seawater are maintained over time as the sea urchins recovered from BSUD. Because both aquaria are equipped with UV lamp housings that function to eliminate live microbes from the seawater, this suggests that the animals release bacteria from their surfaces that impact the aquarium microbiome rather than the reverse. Based on our practices of sea urchin care in which each shipment of sea urchins is housed in different aquaria, the composition of the microbiome of the sea urchins that arrive in a given shipment may determine or greatly influence the microbial composition of the aquarium. Clearly, this variation among sea urchins in different aquaria is maintained to some extent even though the microbiome undergoes major changes when sea urchins are transferred from their natural environment to an aquarium. For example, less than 10% of ASVs identified in the microbiome of spines are retained on the variegated sea urchin, *Lytechinus variegatus*, when they are moved from Biscayne Bay, Florida to a laboratory environment (Wessel et al. 2022). Although the pen/strep treatments that all sea urchins receive upon arrival may also alter the surface microbiome, this treatment improves survival after shipping and has been reported to improve survival of a lesion syndrome disease similar to BSUD in *Strongylocentrotus intermedius* (Wang et al. 2013a). It is noteworthy that the animals housed in aquarium B that acquired BSUD have been the only shipment to our laboratory in which all sea urchins contracted a disease. This may infer that these particular sea urchins were different in some way, possibly in the composition of the microbiome of one or more of the animals, that resulted in all animals contracting BSUD.

### Biomarkers reveal microbial associations with disease and health

Culturing marine bacteria poses challenges because few microbes in a sample will grow on marine media, thereby making it difficult to identify causative agents of disease. Here, we opted to characterize the microbiome associated with BSUD using high throughput sequencing to capture the breadth of microbes associated with the disease. Because bacteria were not cultured, we did not identify a causative agent through reinfection experiments and addressing Koch’s Postulates. Furthermore, the sequence data did not identify a key underlying pathogen of BSUD, but instead showed that there are many taxa with elevated abundance or taxa that are significantly differentially abundant in the microbiomes of the diseased sea urchins. Notable taxa that we identified include *Colwellia, Leucothrix, Aureispira, Spongiibacteraceae, Roseobacteraceae, Rickettsiaceae, Rhodobacteraceae*, and *Flavobacteriaceae*, which may be involved in BSUD progression based on their elevated relative abundances in the microbiomes of the infected sea urchins. The analysis of the same sequences by the ZymoResearch pipeline also identified many of the same taxa as associated with BSUD, particularly *Colwellia* and *Leucothrix*, which strengthens the likelihood of their association with BSUD. However, increased abundances of certain taxa may not necessarily be pathogenic, and instead may be the outcome of an altered host–microbe interaction (Faust et al. 2015). Alternatively, it is also possible that some taxa with low abundance that are also differentially abundant in the diseased group microbiomes may be involved in BSUD progression, such as species of the genera *Aureispira, Lutimonas*, and *Fusibacter* (Fig. 6). However, our assumption here is that taxa involved in BSUD have elevated abundance. Because there are many taxa with increased relative abundance in the infected sea urchin microbiome samples and there is no dominant taxon identified by either pipeline for these samples, it is feasible that a subset of these bacteria act collectively to cause the BSUD symptoms that we observed. This is conceptually akin to microbial dysbiosis (Sweet et al. 2020).

The taxa identified here as associated with BSUD mostly differ from a previous report that analyzed the microbiome of dissected tissues of the collector sea urchin, *Tripneustes gratilla*, that showed a BSUD symptom of primary spine loss (Brink et al. 2019). However, there are major differences in the taxa that show increased abundance in the microbiomes of infected *T. gratilla*, which included *Agarivorans, Arcobacter, Loktanella*, and *Leisingera*, among others that were not identified for infected *S. purpuratus*. Alternatively, *Leucothrix* and *Rhodobacteraceae* are associated with diseased *S. purpuratus* and *T. gratilla*, which suggests that these taxa either contribute to the disease symptoms or are common opportunists. Furthermore, a causative agent underlying BSUD in *T. gratilla* was not identified, leading to the similar conclusion that a combination of microbial agents underlie BSUD symptoms. Nonetheless, the differences between the taxa that we report here for *S. purpuratus* and the report for *T. gratilla* (Brink et al. 2020) infer that different compositions of microbes may cause similar disease symptoms, even in different sea urchin species.

This analysis of BSUD on *S. purpuratus* based on results from both pipelines demonstrated that the majority of the taxa associated with the microbiomes on the diseased sea urchins are Gram negative. However, it was unexpected that *Vibrio* had a similar relative abundance across all three groups (Fig. 5; Supplementary Data File 3; Figure S7I, Supporting Information). This result is inconsistent with multiple reports of direct associations between *Vibrio* and sea urchin disease (Gilles and Pearse 1986, Li et al. 2000, Wang et al. 2005, 2013a, Ho et al. 2016, Hira and Stensvåg 2022) and suggests that *Vibrio* may not be an essential pathogen that causes BSUD in this study. These findings further support the notion that variations in the microbial composition of the microbiome underlies variations in BSUD symptoms, inferring that BSUD infections may be fundamentally different in different species of sea urchins and also in different populations of the same species of sea urchins located in different regions of the oceans. This notion is supported by the differences between the microbiomes of the recovered and healthy sea urchins, which are housed in different aquaria, and have distinct microbial compositions despite being the same species of sea urchin. This may infer that the local microbial marine environment plus the host interaction with the surface microbiome both act to shape the microbial composition on the echinoid surface.

Taxa that are reduced in abundance in the diseased group samples and are elevated in the recovered group samples may not be involved in causing BSUD, but instead may be indicators of a healthy microbiome on the nondiseased sea urchins. Some taxa that are reduced in abundance in the diseased group samples may be outcompeted by other taxa that contribute to the disease symptoms. Bacteroidetes BD-2, *Desulfotalea*, and *Marinifilaceae* may be the best indicators of a healthy microbiome because they are highly abundant on both recovered and healthy sea urchins from both aquaria (Fig. 5). The ZymoResearch pipeline identified similar taxa as reduced in the diseased group microbiome compared to the recovered and healthy groups, such as *Desulfotalea, Alteromonas*, and *Sulfurimonas* (Supplementary Data File 3; Figure S5, Supporting Information). If these taxa are normally elevated in the surface microbiome when sea urchins are in a nondiseased state, decreases in these taxa may be indicators that sea urchins are undergoing dysbiosis, which may be a preliminary condition that leads to disease. Monitoring the taxa of the surface microbiome in aquaria or in natural environments could be used for predicting disease onset.

Recovery from infectious bacterial diseases in any animal infers the involvement of the immune system. Although we did not investigate the complexities of immune functions in the sea urchins, we suggest that in addition to responding to internal infections, the immune system also acts to regulate host–microbe interactions on the animal surface. For example, CRISPRCas9 gene knockout in sea urchins that prevents the production of several naphthoquinones, including echinochrome A and several spinochromes, alters the microbiome of spines and decreases survival of the sea urchins (Yaguchi et al. 2020, Wessel et al. 2022). Naphthoquinone knock-outs also result in the failure of some larvae to survive in a microbial environment (Wessel et al. 2020). The antimicrobial properties of echinochrome A (Service and Wardlaw 1984, Lebedev et al. 2005, Coates et al. 2018) in red spherule cells of adult coelomocytes (Coates et al. 2018; reviewed in Smith et al. 2018) and in pigment cells in larvae (Ho et al. 2016, Buckley et al. 2017) is consistent with their immune functions and responses to bacterial contact, infections, and injuries (Johnson 1969, Heatfield and Travis 1975, Coffaro and Hinegardner 1977, Allen et al. 2022). Thus, the naphthoquinone knock-outs in adult sea urchins infer the presence of a surface immune system and when it is altered, the surface microbiome changes and may result in dysbiosis. This concept of host–microbe regulation in aquatic organisms is consistent with functions of mucosal innate immunity in aquatic vertebrates (Gomez et al. 2013, Varga et al. 2019).

## Conclusions

Here, we describe sea urchins housed in a single aquarium that all contracted BSUD, which is characterized as a surface infection with the loss of all primary spines, in the absence of discrete lesions. Based on the results from two different analytical pipelines, we identify distinct microbial compositions of the surface microbiomes among diseased, recovered, and healthy sea urchins, which show a correlation between recovery from disease and changes to the surface microbiome of sea urchins. We find that the surface microbiome composition can differ among individual animals or among different populations. This suggests that BSUD may be caused by a variety of combinations of bacteria based on both the local microbial environment and the interactions with the sea urchin surface tissues. Results are consistent with our inability, and that of others, to identify a pathogen or a group of microbes that are consistently associated with BSUD, and infers that many BSUD infections, or even each infection, may be unique.

## Supplementary Material

ftad025_Supplemental_Files
