# Supplementary material for: Bald sea urchin disease shifts the surface microbiome on purple sea urchins in an aquarium
Source: Pathog Dis. 2023 Sep 15;81:ftad025. doi: 10.1093/femspd/ftad025 (PMC10550250; doi:10.1093/femspd/ftad025)
Supplement: ftad025_Supplemental_Files [file ftad025_supplemental_files.zip › Shaw etal Supplementary Data File 2 revised.docx]

Supplementary Data File 2

**Bald sea urchin disease shifts the surface microbiome on purple sea urchins in an aquarium**

Chloe G. Shaw, Christina Pavloudi, Megan A. Barela Hudgell, Ryley S. Crow, Jimmy H. Saw, R. Alexander Pyron, L. Courtney Smith

**Supplementary Figures**

**Fig. S1.** Bacterial gDNA is isolated from all samples collected.

**Fig. S2.** Sufficient sampling depth is reached for each sample.

**Supplementary Tables**

**Table S1.** More gDNA is isolated from sea urchin surfaces than from seawater samples

**Table S2.** Sequencing read processing summary

**Table S3.** Most abundant phyla in surface microbiome samples

**Table S4.** Most abundant phyla in the seawater samples

**Table S5.** Relative abundance of the most abundant genera in the microbiomes collected from sea urchins

**Table S6.** Relative abundance of most abundant genera in the seawater samples

**Table S7.** Taxa with significantly different abundances in the microbiomes on sea urchins

**Supplementary Video Legend**

**Video 1.** A sea urchin shows initial symptoms of BSUD


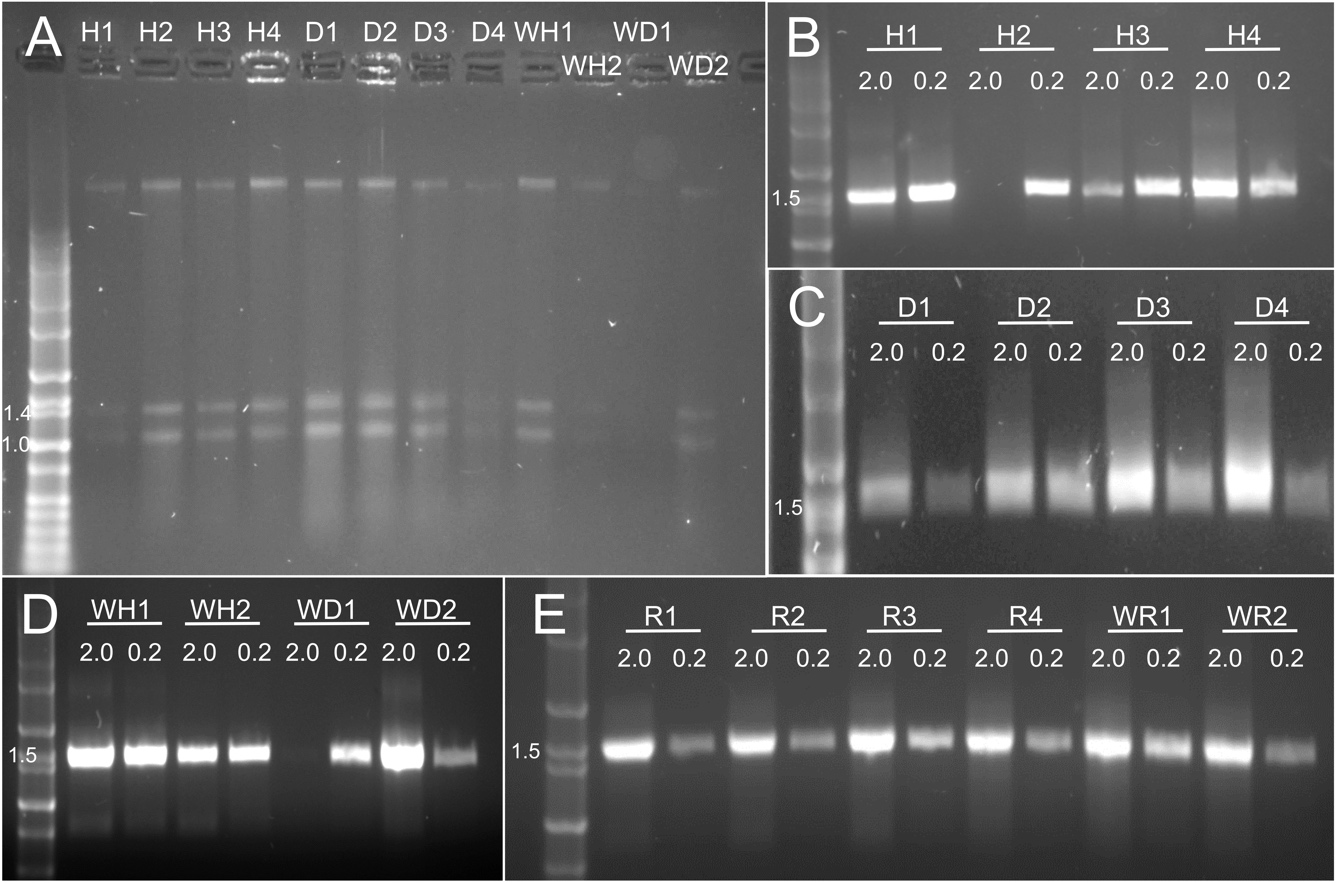


**Fig. S1.** Bacterial gDNA is isolated from all samples collected. **(A)** Selected samples were isolated using the method modified from Turner et al. (2014) and show large, non-degraded gDNA of > 10 kb. The smaller bands of 1.0 kb and 1.4 kb are consistent with rRNA sizes and are likely a mixture from sea urchin and bacteria. The gDNA samples were isolated from healthy sea urchins from aquarium A (H1-H4), diseased sea urchins from aquarium B (D1-D4) as well as seawater samples from aquarium A (WH1, WH2) and aquarium B (WD1, WD2). PCR amplicons of 1.5 kb indicate the presence of the 16S rRNA gene and confirm that bacterial gDNA is present in samples from **(B)** the healthy sea urchins (H1-H4), **(C)** the diseased sea urchins (D1-D4), **(D)** the seawater samples from aquarium A (WH1, WH2) and aquarium B (WD1, WD2), and **(E)** the recovered sea urchins (R1-R4), and seawater samples from aquarium B (WR1, WR2). Lanes with missing bands are likely the result of technical errors. The gDNA samples were separated by electrophoresis on a 0.75% agarose gel and amplicons were separated on a 0.8% agarose gel, both with ethidium bromide, and all samples were imaged on a UV imager (see the main paper for details). The standard DNA size marker used for all gels is the Hi-Lo DNA marker (Bionexus).

**
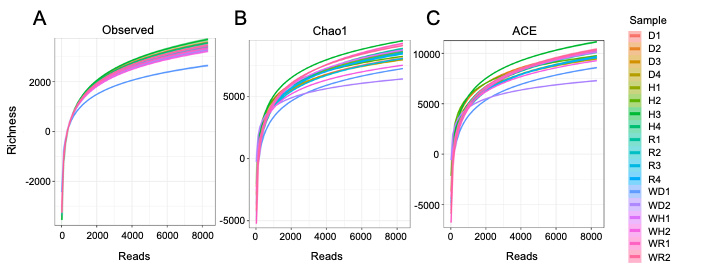
**

**Fig. S2.** Sufficient sampling depth is reached for each sample. Rarefaction curves for all samples reach a plateau for each analytical index **(A-C)**, indicating that sufficient numbers of sequences were acquired for each sample to allow fair comparisons among samples in subsequent analyses.

**Supplementary Tables**

**Table S1.** More gDNA is isolated from sea urchin surfaces than from seawater samples

| **Group^1^** | **Total gDNA (ng/µl)** | **Average gDNA per sample (ng/µl)** | **Fold difference of gDNA^2^** |
| --- | --- | --- | --- |
| D | 437.0 | 109.25 | 2.94 |
| WD | 74.2 | 37.1 |  |
| R | 5,666.9 | 1,416.7 | 34.85 |
| WR | 81.3 | 40.65 |  |
| H | 304.1 | 76.0 | 1.07 |
| WH | 142.1 | 71.05 |  |

^1^Abbreviations are defined in the legend to Figure S1 and in the materials and methods section in the main paper.

^2^Fold difference in gDNA isolated from sea urchin surfaces compared to the seawater samples for that group.

**Table S2.** Sequencing read processing summary

| **Sample name^1^** | **Initial reads** | **Filtered reads** | **Forward denoised reads** | **Reverse denoised reads** | **Merged reads** | **Non-chimeric reads** |
| --- | --- | --- | --- | --- | --- | --- |
| D1 | 62041 | 53550 | 50737 | 51861 | 39098 | 14633 |
| D2 | 59327 | 52024 | 47960 | 49910 | 34612 | 11500 |
| D3 | 75054 | 64402 | 61194 | 62418 | 46355 | 15663 |
| D4 | 73440 | 63520 | 60793 | 61689 | 48792 | 14935 |
| WD1 | 51745 | 44029 | 39384 | 41467 | 26509 | 12821 |
| WD2 | 51975 | 45231 | 42110 | 43563 | 31123 | 9607 |
| R1 | 61269 | 51932 | 49079 | 49967 | 37565 | 16126 |
| R2 | 60408 | 52664 | 50034 | 50636 | 38726 | 15887 |
| R3 | 74635 | 64853 | 62084 | 62758 | 51486 | 15637 |
| R4 | 79356 | 69293 | 65266 | 66838 | 50461 | 19957 |
| WR1 | 74405 | 63647 | 59956 | 61157 | 45512 | 19169 |
| WR1 | 53088 | 45367 | 41797 | 43518 | 30835 | 13397 |
| H1 | 70784 | 60532 | 56611 | 58006 | 41897 | 17441 |
| H2 | 76183 | 65947 | 62304 | 63728 | 47109 | 17543 |
| H3 | 63792 | 53973 | 50921 | 51997 | 37271 | 14926 |
| H4 | 64526 | 54091 | 50916 | 52074 | 37645 | 15260 |
| WH1 | 69870 | 59633 | 56229 | 57318 | 41962 | 16636 |
| WH1 | 31474 | 27540 | 24756 | 26138 | 16857 | 8489 |
| Blank | 68225 | 57547 | 55266 | 56273 | 47114 | 13670 |
| Total | 1221597 | 1049775 | 987397 | 1011316 | 750929 | 283297 |

^1^Abbreviations are defined in the legend to Figure S1 and in the Materials and Methods section in the main paper.

**Table S3.** Most abundant phyla in surface microbiome samples

| **Phylum^1^** | **D1^2^** | **D2** | **D3** | **D4** | **R1** | **R2** | **R3** | **R4** | **H1** | **H2** | **H3** | **H4** |
| --- | --- | --- | --- | --- | --- | --- | --- | --- | --- | --- | --- | --- |
| Proteobacteria | 64.5^3^ | 62 | 66.34 | 59.29 | 57.69 | 60.73 | 34.92 | 49.6 | 54.17 | 45.42 | 46.49 | 54.03 |
| Bacteroidota | 19.5 | 24.59 | 18.03 | 22.15 | 14.79 | 12.64 | 19.69 | 16.1 | 24.32 | 27.33 | 27.6 | 24.12 |
| Verrucomicrobiota | 5.06 | 4.65 | 4.35 | 4.19 | 8.26 | 6.47 | 9.92 | 8.55 | 4.52 | 4.33 | 5.11 | 3.82 |
| Planctomycetota | 3.14 | 2.08 | 3.09 | 2.65 | 4.09 | 4.49 | 5.36 | 5.95 | 4.01 | 1.53 | 3.34 | 4.28 |
| Desulfobacterota | 0.49 | 0.51 | 0.62 | 0.86 | 2.57 | 2.59 | 8.38 | 4.82 | 3.92 | 5.55 | 8.04 | 4.24 |
| Firmicutes | 0.6 | 1.05 | 0.6 | 1.52 | 2.43 | 2.7 | 5.76 | 3.18 | 1.82 | 10.96 | 1.93 | 1.59 |
| Campylobacterota | 2.13 | 1.81 | 2.45 | 4.29 | 1.4 | 2.14 | 6.22 | 2.41 | 0.67 | 0.69 | 0.75 | 0.75 |
| Spirochaetota | 0.04 | 0.01 | 0.06 | 0.04 | 2.11 | 1.4 | 2.94 | 1.45 | 0.99 | 1.63 | 1.83 | 1.38 |
| Bdellovibrionota | 0.78 | 0.57 | 1 | 0.92 | 1.25 | 1.13 | 1.97 | 1.39 | 0.49 | 0.41 | 0.89 | 0.69 |
| Patescibacteria | 0.32 | 0.23 | 0.5 | 0.4 | 1.7 | 1.53 | 1.63 | 1.97 | 1.04 | 0.39 | 0.5 | 1.01 |
| Myxococcota | 0.39 | 0.33 | 0.38 | 0.36 | 0.57 | 0.84 | 0.3 | 0.74 | 0.49 | 0.18 | 0.32 | 0.45 |
| Actinobacteriota | 0.61 | 0.59 | 0.79 | 0.74 | 0.31 | 0.4 | 0.21 | 0.34 | 0.63 | 0.22 | 0.32 | 0.62 |
| Crenarchaeota | 0.08 | 0.04 | 0.07 | 0.07 | 0.66 | 1.09 | 0.41 | 0.81 | 0.51 | 0.27 | 0.42 | 0.5 |
| Cyanobacteria | 0.55 | 0.22 | 0.46 | 1.3 | 0.4 | 0.2 | 0.46 | 0.19 | 0.55 | 0.19 | 0.36 | 0.95 |
| Bacteria | 0.08 | 0.21 | 0.05 | 0.05 | 0.43 | 0.22 | 0.44 | 0.66 | 0.31 | 0.24 | 0.52 | 0.36 |
| Halobacterota | 0 | 0 | 0 | 0.01 | 0.01 | 0.01 | 0 | 0 | 0.01 | 0 | 0 | 0.01 |
| Chloroflexi | 0.93 | 0.48 | 0.56 | 0.55 | 0.13 | 0.19 | 0.07 | 0.14 | 0.05 | 0.02 | 0.03 | 0 |
| Entotheonellaeota | 0.01 | 0.01 | 0.01 | 0.03 | 0.11 | 0.15 | 0.1 | 0.2 | 0.53 | 0.17 | 0.4 | 0.36 |
| Acidobacteriota | 0.08 | 0.01 | 0.04 | 0.02 | 0.32 | 0.34 | 0.14 | 0.35 | 0.09 | 0.03 | 0.05 | 0.04 |
| Marinimicrobia (SAR406 clade) | 0.01 | 0.01 | 0.01 | 0.01 | 0.21 | 0.05 | 0.67 | 0.21 | 0.07 | 0.08 | 0.59 | 0.17 |
| Nanoarchaeota | 0.2 | 0.18 | 0.17 | 0.21 | 0.19 | 0.13 | 0.23 | 0.19 | 0.05 | 0.08 | 0.04 | 0.09 |
| Fusobacteriota | 0.27 | 0.24 | 0.26 | 0.22 | 0.04 | 0.07 | 0.04 | 0.1 | 0.12 | 0.09 | 0.12 | 0.1 |

^1^Phyla with an relative percent abundance of > 0.1% for at least one sample.

^2^Abbreviations are defined in the legend to Figure S1 (above) and in the Materials and Methods section in the main paper.

^2^Percent relative abundance per sample.

**Table S4.** Most abundant phyla in the seawater samples

| **Phylum^1^** | **WD1^2^** | **WD2** | **WR1** | **WR2** | **WH1** | **WH2** |
| --- | --- | --- | --- | --- | --- | --- |
| Proteobacteria | 56.52^3^ | 61.98 | 57.84 | 62.32 | 54.2 | 58.83 |
| Bacteroidota | 21.73 | 24.07 | 13.94 | 12.19 | 22.76 | 23.76 |
| Verrucomicrobiota | 5.98 | 5.49 | 7.03 | 6.88 | 5 | 5.66 |
| Planctomycetota | 5.06 | 2.89 | 6.35 | 5.26 | 4.95 | 3.4 |
| Desulfobacterota | 1.06 | 0.34 | 2.95 | 2.04 | 3.47 | 1.96 |
| Firmicutes | 0.22 | 0.61 | 1.7 | 1.87 | 1.41 | 0.73 |
| Campylobacterota | 0.81 | 1.64 | 1.87 | 1.83 | 0.69 | 0.47 |
| Spirochaetota | 0.07 | 0.01 | 1.19 | 1.18 | 1.06 | 0.34 |
| Bdellovibrionota | 0.91 | 0.54 | 1.06 | 0.93 | 0.62 | 0.44 |
| Patescibacteria | 0.17 | 0.16 | 1.39 | 1.53 | 0.89 | 0.24 |
| Myxococcota | 1.04 | 0.15 | 0.93 | 0.68 | 0.91 | 0.94 |
| Actinobacteriota | 0.33 | 0.84 | 0.62 | 0.71 | 0.4 | 0.35 |
| Crenarchaeota | 0.16 | 0.04 | 1.03 | 0.75 | 0.82 | 0.47 |
| Cyanobacteria | 0.17 | 0.17 | 0.18 | 0.27 | 0.65 | 0.66 |
| Bacteria | 0.23 | 0.26 | 0.39 | 0.26 | 0.34 | 0.24 |
| Halobacterota | 4.32 | 0.01 | 0 | 0 | 0.01 | 0.02 |
| Chloroflexi | 0.26 | 0.57 | 0.18 | 0.11 | 0.1 | 0.02 |
| Entotheonellaeota | 0 | 0 | 0.15 | 0.13 | 0.69 | 0.4 |
| Acidobacteriota | 0 | 0.03 | 0.43 | 0.56 | 0.1 | 0.2 |
| Marinimicrobia (SAR406 clade) | 0.02 | 0 | 0.06 | 0.03 | 0.09 | 0 |
| Nanoarchaeota | 0.1 | 0.05 | 0.08 | 0.04 | 0.06 | 0.02 |
| Fusobacteriota | 0.08 | 0.08 | 0.01 | 0.03 | 0.09 | 0.02 |

^1^Phyla with an average relative percent abundance across all samples of > 0.1%

^2^Abbreviations are defined in the legend to Figure S1 (above and in the Materials and Methods section in the main paper.

^3^Percent relative abundance per sample

**Table S5.** Relative abundance of the most abundant genera in the microbiomes collected from sea urchins

| **Taxa^1^** | **D1^2^** | **D2** | **D3** | **D4** | **R1** | **R2** | **R3** | **R4** | **H1** | **H2** | **H3** | **H4** |
| --- | --- | --- | --- | --- | --- | --- | --- | --- | --- | --- | --- | --- |
| *Psychromonas* | 6.6^3^ | 6.62 | 7.54 | 7.73 | 9.56 | 9.64 | 6.85 | 8.43 | 6.84 | 7.22 | 6.96 | 6.76 |
| *Vibrio* | 3.42 | 2.63 | 5.87 | 4.44 | 2.29 | 2.27 | 3.67 | 2.17 | 2.96 | 9.67 | 4.72 | 3.36 |
| *Colwellia* | 6.36 | 7.87 | 9.16 | 6.37 | 0.96 | 1.37 | 0.64 | 1.16 | 1.42 | 1.41 | 1.96 | 1.59 |
| Rhodobacteraceae | 2.63 | 6.25 | 2.67 | 2.04 | 2.12 | 2.49 | 1.19 | 2.27 | 2.65 | 1.06 | 1.72 | 2.58 |
| *Leucothrix* | 4.43 | 5.51 | 4.67 | 3.88 | 0.51 | 0.52 | 0.28 | 0.42 | 2.06 | 0.76 | 1.01 | 1.56 |
| Marinifilaceae | 1.56 | 1.28 | 1.62 | 1.55 | 2.35 | 2.36 | 4.62 | 2.48 | 3.5 | 5.13 | 4.79 | 3.8 |
| *Desulfotalea* | 0.32 | 0.38 | 0.35 | 0.59 | 1.17 | 1.03 | 3.42 | 1.75 | 2.79 | 4.95 | 4.48 | 2.72 |
| Bacteroidia | 0.8 | 0.55 | 0.52 | 1.11 | 2.54 | 1.35 | 3.25 | 2.18 | 2.46 | 3.5 | 3.07 | 2.39 |
| Gammaproteobacteria | 1.31 | 1.61 | 1.67 | 1.38 | 2.09 | 1.95 | 1.1 | 2.18 | 0.66 | 0.75 | 0.71 | 1.11 |
| Spongiibacteraceae | 1.68 | 1.3 | 1.69 | 1.58 | 2.33 | 2.44 | 1.3 | 2.07 | 0.52 | 0.28 | 0.37 | 0.54 |
| *Pseudoalteromonas* | 0.86 | 0.52 | 1.87 | 0.82 | 0.76 | 1.17 | 0.85 | 1.15 | 1.58 | 1.81 | 2.47 | 2.96 |
| *Roseimarinus* | 1.1 | 0.76 | 1.09 | 1.44 | 0.59 | 0.84 | 2.18 | 1.46 | 1.55 | 4.17 | 2.41 | 1.21 |
| Bacteroidetes BD2-2 | 0.34 | 0.19 | 0.26 | 0.34 | 0.96 | 0.62 | 1.56 | 1.3 | 2.01 | 2.1 | 3.65 | 2.52 |
| *Ruegeria* | 1.41 | 1.48 | 0.95 | 0.75 | 0.96 | 1.22 | 0.66 | 0.93 | 0.34 | 0.18 | 0.33 | 0.39 |
| Erwiniaceae | 4.05 | 2.01 | 3.54 | 5.09 | 1.36 | 0.69 | 1.05 | 0.25 | 0.33 | 0.56 | 0.99 | 1.26 |
| *Lutibacter* | 1.12 | 1.45 | 1.7 | 2.95 | 0.34 | 0.29 | 0.28 | 0.34 | 0.62 | 1.81 | 0.64 | 0.53 |
| *Pseudahrensia* | 0.69 | 0.76 | 0.66 | 0.62 | 0.9 | 1.48 | 0.4 | 0.75 | 1.94 | 0.96 | 1.06 | 1.96 |
| Alteromonadaceae | 0.39 | 0.83 | 0.45 | 0.34 | 1.94 | 2.58 | 1.39 | 2.3 | 0.32 | 0.31 | 0.35 | 0.48 |
| Alphaproteobacteria | 1.1 | 1.03 | 1 | 0.87 | 1.61 | 1.74 | 0.53 | 1.65 | 0.83 | 0.41 | 0.5 | 0.85 |
| vadinHA21 | 0.24 | 0.36 | 0.29 | 0.41 | 0.97 | 0.77 | 1.62 | 2.01 | 1.51 | 1.31 | 1.85 | 1.44 |
| NS10 marine group | 2.03 | 1.69 | 1.78 | 4.63 | 0.1 | 0.18 | 0.89 | 0.12 | 0.37 | 1.86 | 1.51 | 0.84 |

^1^Taxa were selected that had an average relative abundance across all samples > 1%. The taxa were identified as species and the lowest known taxonomic level for each taxon is listed.

^2^Abbreviations are defined in the legend to Figure S1 (above) and in the Materials and Methods section in the main paper.

^3^Percent relative abundance per sample

**Table S6.** Relative abundance of the most abundant genera in the seawater samples

| **Taxa^1^** | **WD1^2^** | **WD2** | **WR1** | **WR2** | **WH1** | **WH2** |
| --- | --- | --- | --- | --- | --- | --- |
| *Psychromonas* | 2.44^3^ | 4.71 | 8.89 | 11.33 | 8.94 | 6.08 |
| *Vibrio* | 3.74 | 2.28 | 1.97 | 2.13 | 3.23 | 5.54 |
| *Colwellia* | 2.47 | 5.56 | 1.24 | 1.38 | 1.94 | 1.67 |
| Rhodobacteraceae | 3.21 | 7.17 | 3.41 | 3.58 | 2.06 | 2.52 |
| *Leucothrix* | 7.05 | 11.64 | 0.71 | 0.73 | 1.51 | 0.82 |
| Marinifilaceae | 0.34 | 0.8 | 1.98 | 1.64 | 3.82 | 1.89 |
| *Desulfotalea* | 0.67 | 0.19 | 1.14 | 1.04 | 2.38 | 1.33 |
| Bacteroidia | 0.33 | 0.77 | 1.12 | 0.74 | 2.4 | 1.36 |
| Gammaproteobacteria | 0.94 | 1.46 | 2.59 | 2.87 | 0.81 | 1.13 |
| Spongiibacteraceae | 0.35 | 1.55 | 2.9 | 2.94 | 0.65 | 0.32 |
| *Pseudoalteromonas* | 0.13 | 0.3 | 1.27 | 1.13 | 1.78 | 1.81 |
| *Roseimarinus* | 0.29 | 0.44 | 0.8 | 0.51 | 1.07 | 0.52 |
| Bacteroidetes BD2-2 | 0.09 | 0.12 | 1.23 | 1.28 | 2.39 | 1.23 |
| *Ruegeria* | 5.65 | 2.13 | 1.71 | 1.47 | 0.34 | 1.15 |
| Erwiniaceae | 0.02 | 0.64 | 0 | 0 | 0.01 | 0.08 |
| *Lutibacter* | 3.25 | 1.28 | 0.3 | 0.38 | 0.43 | 2.03 |
| *Pseudahrensia* | 0.55 | 1.07 | 1.23 | 1.13 | 1.62 | 1.59 |
| Alteromonadaceae | 0.4 | 0.75 | 2.28 | 2.38 | 0.77 | 0.66 |
| Alphaproteobacteria | 0.31 | 0.79 | 2.06 | 1.91 | 1.08 | 0.62 |
| vadinHA21 | 0.42 | 0.23 | 1.07 | 0.98 | 1.6 | 1.61 |
| NS10 marine group | 0.78 | 1.23 | 0.05 | 0.06 | 0.33 | 0.13 |

^1^Taxa were selected that had an average relative abundance across all samples > 1%. The taxa were identified as species and the lowest known taxonomic level for each taxon is listed.

^2^Abbreviations are defined in the legend to Figure S1 and in the materials and methods section in the main paper.

^3^Percent relative abundance per sample

**Table S7.** Taxa with significantly different abundances in the microbiomes on sea urchins^1^

| **Group** | **ef_lda^2^** | ***p* value** | **padj** | **Taxonomy^3^** |
| --- | --- | --- | --- | --- |
| Diseased | 3.32 | 0.003 | 0.003 | Amoebophilaceae |
| Diseased | 3.30 | 0.024 | 0.024 | *Aureispira* |
| Diseased | 3.29 | 0.003 | 0.003 | Spongiibacteraceae |
| Diseased | 3.28 | 0.004 | 0.004 | Candidatus *Berkiella* |
| Diseased | 3.25 | 0.002 | 0.002 | Saprospiraceae |
| Diseased | 3.23 | 0.001 | 0.001 | *Reichenbachiella* |
| Diseased | 3.22 | 0.001 | 0.001 | *Luteibaculum* |
| Diseased | 3.22 | 0.001 | 0.001 | Rickettsiaceae |
| Diseased | 3.21 | 0.000 | 0.000 | *Pseudoteredinibacter* |
| Diseased | 3.21 | 0.001 | 0.001 | *Aureispira* |
| Diseased | 3.19 | 0.003 | 0.003 | *Halocynthiibacter* |
| Diseased | 3.18 | 0.032 | 0.032 | Gammaproteobacteria EC3 |
| Diseased | 3.17 | 0.027 | 0.027 | *Lutimonas* |
| Diseased | 3.17 | 0.001 | 0.001 | Anaerolineae, SBR1031, A4b |
| Diseased | 3.15 | 0.005 | 0.005 | *Colwellia* |
| Diseased | 3.14 | 0.007 | 0.007 | *Roseobacter* |
| Diseased | 3.14 | 0.016 | 0.016 | Rhodobacteraceae |
| Diseased | 3.13 | 0.001 | 0.001 | Saccharospirillaceae |
| Diseased | 3.12 | 0.001 | 0.001 | *Roseimarinus* |
| Diseased | 3.11 | 0.005 | 0.005 | Flavobacteriaceae |
| Diseased | 3.10 | 0.003 | 0.003 | *Haloferula* |
| Diseased | 3.10 | 0.001 | 0.001 | Planctomycetota vadinHA49 |
| Diseased | 3.10 | 0.004 | 0.004 | Flavobacteriaceae |
| Diseased | 3.09 | 0.001 | 0.001 | *Fusibacter* |
| Diseased | 3.08 | 0.000 | 0.000 | Arcobacteraceae |
| Diseased | 3.08 | 0.001 | 0.001 | *Leucothrix* |
| Diseased | 3.08 | 0.014 | 0.014 | Cryomorphaceae |
| Diseased | 3.08 | 0.001 | 0.001 | *Halocynthiibacter* |
| Diseased | 3.08 | 0.003 | 0.003 | *Planctomicrobium* |
| Diseased | 3.06 | 0.001 | 0.001 | Anaerolineae SBR1031 A4b |
| Diseased | 3.05 | 0.008 | 0.008 | Halieaceae |
| Diseased | 3.05 | 0.001 | 0.001 | Spongiibacteraceae BD1-7 clade |
| Diseased | 3.03 | 0.001 | 0.001 | Rickettsiales |
| Diseased | 3.03 | 0.002 | 0.002 | Verrucomicrobiales DEV007 |
| Diseased | 3.02 | 0.002 | 0.002 | Bdellovibrionaceae OM27 clade |
| Diseased | 3.02 | 0.001 | 0.001 | *Donghicola* |
| Diseased | 3.02 | 0.000 | 0.000 | *Poseidonibacter* |
| Diseased | 3.02 | 0.001 | 0.001 | Chitinophagales 37-13 |
| Diseased | 3.02 | 0.003 | 0.003 | *Rhodopirellula* |
| Diseased | 3.00 | 0.002 | 0.002 | *Amylibacter* |
| Diseased | 3.00 | 0.001 | 0.001 | Rickettsiaceae |
| Diseased | 3.00 | 0.003 | 0.003 | Granulosicoccus |
| Healthy | 3.30 | 0.001 | 0.001 | *Rubidimonas* |
| Healthy | 3.30 | 0.002 | 0.002 | *Jannaschia* |
| Healthy | 3.27 | 0.004 | 0.004 | *Clostridia* |
| Healthy | 3.27 | 0.003 | 0.003 | *Draconibacterium* |
| Healthy | 3.26 | 0.001 | 0.001 | Terasakiellaceae |
| Healthy | 3.25 | 0.002 | 0.002 | *Ulvibacter* |
| Healthy | 3.25 | 0.001 | 0.001 | Phycisphaerales AKAU3564 sediment group |
| Healthy | 3.23 | 0.000 | 0.000 | Flavobacteriaceae |
| Healthy | 3.22 | 0.001 | 0.001 | Bacteroidia |
| Healthy | 3.22 | 0.001 | 0.001 | Chloroplast |
| Healthy | 3.21 | 0.001 | 0.001 | Hyphococcus |
| Healthy | 3.21 | 0.000 | 0.000 | Hyphomonadaceae |
| Healthy | 3.18 | 0.001 | 0.001 | Candidatus *Entotheonella* |
| Healthy | 3.16 | 0.001 | 0.001 | Bacteroidetes BD2-2 |
| Healthy | 3.14 | 0.001 | 0.001 | *Devosia* |
| Healthy | 3.13 | 0.001 | 0.001 | Bdellovibrionaceae OM27 clade |
| Healthy | 3.13 | 0.006 | 0.006 | *Clostridia* vadinBB60 group |
| Healthy | 3.12 | 0.001 | 0.001 | Kiritimatiellaceae MSBL3 |
| Healthy | 3.11 | 0.001 | 0.001 | *Desulfotalea* |
| Healthy | 3.11 | 0.021 | 0.021 | Marinifilaceae |
| Healthy | 3.11 | 0.001 | 0.001 | *Shewanella* |
| Healthy | 3.11 | 0.000 | 0.000 | *Pseudahrensia* |
| Healthy | 3.10 | 0.008 | 0.008 | *Clostridia* vadinBB60 group |
| Healthy | 3.10 | 0.001 | 0.001 | *Pseudahrensia* |
| Healthy | 3.10 | 0.004 | 0.004 | *Desulfobulbus* |
| Healthy | 3.09 | 0.001 | 0.001 | *Roseobacter* clade  *Marinomonas* lineage |
| Healthy | 3.08 | 0.001 | 0.001 | *Agarivorans* |
| Healthy | 3.07 | 0.003 | 0.003 | Planctomycetota OM190 |
| Healthy | 3.07 | 0.003 | 0.003 | *Spongiibacterium* |
| Healthy | 3.07 | 0.002 | 0.002 | Lentisphaeria P.palmC41 |
| Healthy | 3.06 | 0.003 | 0.003 | Candidatus *Riegeria* |
| Healthy | 3.05 | 0.001 | 0.001 | Flavobacteriaceae |
| Healthy | 3.04 | 0.001 | 0.001 | Chitinophagales |
| Healthy | 3.04 | 0.001 | 0.001 | Planctomycetota OM190 |
| Healthy | 3.02 | 0.001 | 0.001 | Lachnospiraceae |
| Healthy | 3.02 | 0.001 | 0.001 | *Rhodopirellula* |
| Healthy | 3.02 | 0.006 | 0.006 | *Halioglobus* |
| Healthy | 3.01 | 0.007 | 0.007 | *Clostridia* vadinBB60 group |
| Healthy | 3.01 | 0.004 | 0.004 | Christensenellaceae |
| Healthy | 3.01 | 0.005 | 0.005 | *Roseimarinus* |
| Healthy | 3.01 | 0.004 | 0.004 | *Psychroflexus* |
| Healthy | 3.00 | 0.001 | 0.001 | *Devosia* |
| Recovered | 3.44 | 0.001 | 0.001 | *Desulfovibrio* |
| Recovered | 3.41 | 0.001 | 0.001 | Kiritimatiellae WCHB1-41 |
| Recovered | 3.26 | 0.008 | 0.008 | *Desulfovibrio* |
| Recovered | 3.24 | 0.000 | 0.000 | Hyphomonadaceae |
| Recovered | 3.22 | 0.009 | 0.009 | Kiritimatiellaceae R76-B128 |
| Recovered | 3.22 | 0.001 | 0.001 | *Clostridia* |
| Recovered | 3.20 | 0.001 | 0.001 | *Thalassotalea* |
| Recovered | 3.19 | 0.002 | 0.002 | Hungateiclostridiaceae |
| Recovered | 3.19 | 0.005 | 0.005 | *Aestuariicella* |
| Recovered | 3.18 | 0.001 | 0.001 | Bacteriovoracaceae |
| Recovered | 3.18 | 0.000 | 0.000 | Spongiibacteraceae |
| Recovered | 3.18 | 0.001 | 0.001 | *Rubritalea* |
| Recovered | 3.17 | 0.000 | 0.000 | Bacteriovoracaceae |
| Recovered | 3.17 | 0.001 | 0.001 | *Desulforhopalus* |
| Recovered | 3.13 | 0.001 | 0.001 | Gracilibacteria JGI 0000069-P22 |
| Recovered | 3.13 | 0.001 | 0.001 | Lachnospiraceae |
| Recovered | 3.12 | 0.001 | 0.001 | Micavibrionaceae |
| Recovered | 3.11 | 0.001 | 0.001 | Spirochaetota MVP-15 |
| Recovered | 3.11 | 0.001 | 0.001 | Phycisphaerales AKAU3564 sediment group |
| Recovered | 3.10 | 0.002 | 0.002 | *Coxiella* |
| Recovered | 3.10 | 0.001 | 0.001 | Christensenellaceae |
| Recovered | 3.10 | 0.001 | 0.001 | Absconditabacteriales (SR1) |
| Recovered | 3.09 | 0.002 | 0.002 | Rickettsiales SM2D12 |
| Recovered | 3.08 | 0.002 | 0.002 | Bacteroidales vadinHA21 |
| Recovered | 3.07 | 0.001 | 0.001 | Desulfobacteraceae |
| Recovered | 3.07 | 0.008 | 0.008 | Rickettsiales |
| Recovered | 3.07 | 0.002 | 0.002 | Rubinisphaeraceae |
| Recovered | 3.06 | 0.004 | 0.004 | *Coxiella* |
| Recovered | 3.05 | 0.002 | 0.002 | Gammaproteobacteria |
| Recovered | 3.04 | 0.001 | 0.001 | Bdellovibrionaceae OM27 clade |
| Recovered | 3.04 | 0.002 | 0.002 | Gammaproteobacteria |
| Recovered | 3.04 | 0.003 | 0.003 | *Roseibacillus* |
| Recovered | 3.03 | 0.003 | 0.003 | Acidobacteriae PAUC26f |
| Recovered | 3.02 | 0.005 | 0.005 | *Clostridia* |
| Recovered | 3.02 | 0.001 | 0.001 | *Clostridia* |
| Recovered | 3.02 | 0.001 | 0.001 | Chitinophagales |
| Recovered | 3.02 | 0.001 | 0.001 | *Vibrio* |
| Recovered | 3.01 | 0.001 | 0.001 | *Oceanicoccus* |
| Recovered | 3.00 | 0.000 | 0.000 | *Aliikangiella* |

^1^Data were generated by LEfSe analysis

^2^Linear Discriminant Analysis; LDA cutoff was set to 3.0.

^3^Identified taxa are ASVs and the lowest known taxonomic classification is listed.

**Video 1 Legend.**  A sea urchin shows initial symptoms of BSUD. The primary spines droop downwards, which only occurs when the muscles at the base of the spines are relaxed. This behavior may be reminiscent of sleep, which has been observed in healthy sea urchins (LC Smith, unpublished), or it may be an outcome of infection and partial destruction of the muscles that attach the spines to the tubercles. In response to an external disturbance (tapping on the aquarium glass), the sea urchin reorients its spines (see Video 1). Detached spines are visible on the aquarium floor, indicating that sea urchins in aquarium B have started to lose their primary spines, which is the key symptom of BSUD.
