# Supplementary material for: Bald sea urchin disease shifts the surface microbiome on purple sea urchins in an aquarium
Source: Pathog Dis. 2023 Sep 15;81:ftad025. doi: 10.1093/femspd/ftad025 (PMC10550250; doi:10.1093/femspd/ftad025)
Supplement: ftad025_Supplemental_Files [file ftad025_supplemental_files.zip › Shaw etal Supplementary Data File 3 revised.docx]

Supplementary Data File 3

**Results of sequence analysis by the ZymoResearch Pipeline**

**Bald sea urchin disease shifts the surface microbiome on purple sea urchins in an aquarium**

Chloe G. Shaw, Christina Pavloudi, Megan A. Barela Hudgell, Ryley S. Crow, Jimmy H. Saw, R. Alexander Pyron, L. Courtney Smith

**Supplementary Figures**

**Fig. S1.** Sufficient sampling depth is reached for each sample.

**Fig. S2.** Alpha diversity of the microbiomes is not different among groups.

**Fig. S3.** The microbial compositions are different among the microbiomes of the three groups of sea urchins.

**Fig. S4.** The most abundant phyla in the surface microbiomes are Proteobacteria and Bacteroidota.

**Fig. S5.** The most abundant taxa are different in the microbiome samples collected from the three groups of sea urchins.

**Fig. S6.** Taxa have different abundances in microbiomes from samples collected from diseased sea urchins compared to recovered sea urchins and from recovered sea urchins compared to healthy sea urchins.

**Fig. S7.** Some taxa show differences in the microbiomes of the diseased compared to the recovered groups, plus differences based on different shipments of sea urchins housed in two aquaria.

**Supplementary Tables**

**Table S1.** ASVs are identified in each sample

**Table S2.** Phyla of highest abundance in surface microbiome samples

**Table S3.** Bacterial taxa of > 2% relative abundance in each sample group

**Table S4.** Biomarker taxa of significantly different abundances and large effect size

**Table S5.** Biomarker genera of significantly different abundances and large effect size

**Methods**

**Amplicon sequence analysis by the ZymoResearch pipeline**

The DADA2 pipeline (Callahan et al. 2016) was used to infer amplicon sequence variants (ASVs) from raw reads and to remove sequencing errors and chimeric sequences. Taxonomy assignment was performed using Uclust from Qiime v.1.9.1 (Caporaso et al. 2010) with the curated ZymoResearch Database. Data were rarefied to a depth of 19,050 sequences/sample. Diversity estimates for Chao1, Shannon and Simpson were carried out and visualized using R. Statistical significance of differences among groups for alpha diversity indices was performed using ANOVA (*p* ≤ 0.05). Beta diversity was analyzed by Bray-Curtis distances matrices and plotted using Principal Coordinates Analysis. To evaluate statistical significance among groups for beta diversity, a permutational multivariate analysis of variance (PERMANOVA, *p* ≤ 0.05) using distance matrices was performed with the adonis function (Permutations = 999) with the vegan package (Oksanen et al. 2015) in R. This was followed by the *p* adjust function by the Benjamini and Hochberg (1995) method to correct for errors and to prevent false positives. Taxonomic groups that had significant differences in abundance among different groups were identified by Linear Discriminant Analysis Effect Size analysis (LEfSe) (Segata et al. 2011) using the following parameters: Kruskal-Wallis test alpha value = 0.05, Wilcoxon test alpha value = 0.05, LDA score threshold = 2, multiclass analysis strategy = all-against-all. All other analyses including Taxa2ASV Decomposer plots were performed using internal scripts at ZymoResearch. Absolute abundance was quantified by real-time PCR with a standard curve, which was made with plasmid DNA that contained the 16S rRNA gene. The primers used for sequencing were identical to those used for library preparation (see the main paper for primer sequence information).

**Supplementary Figures**


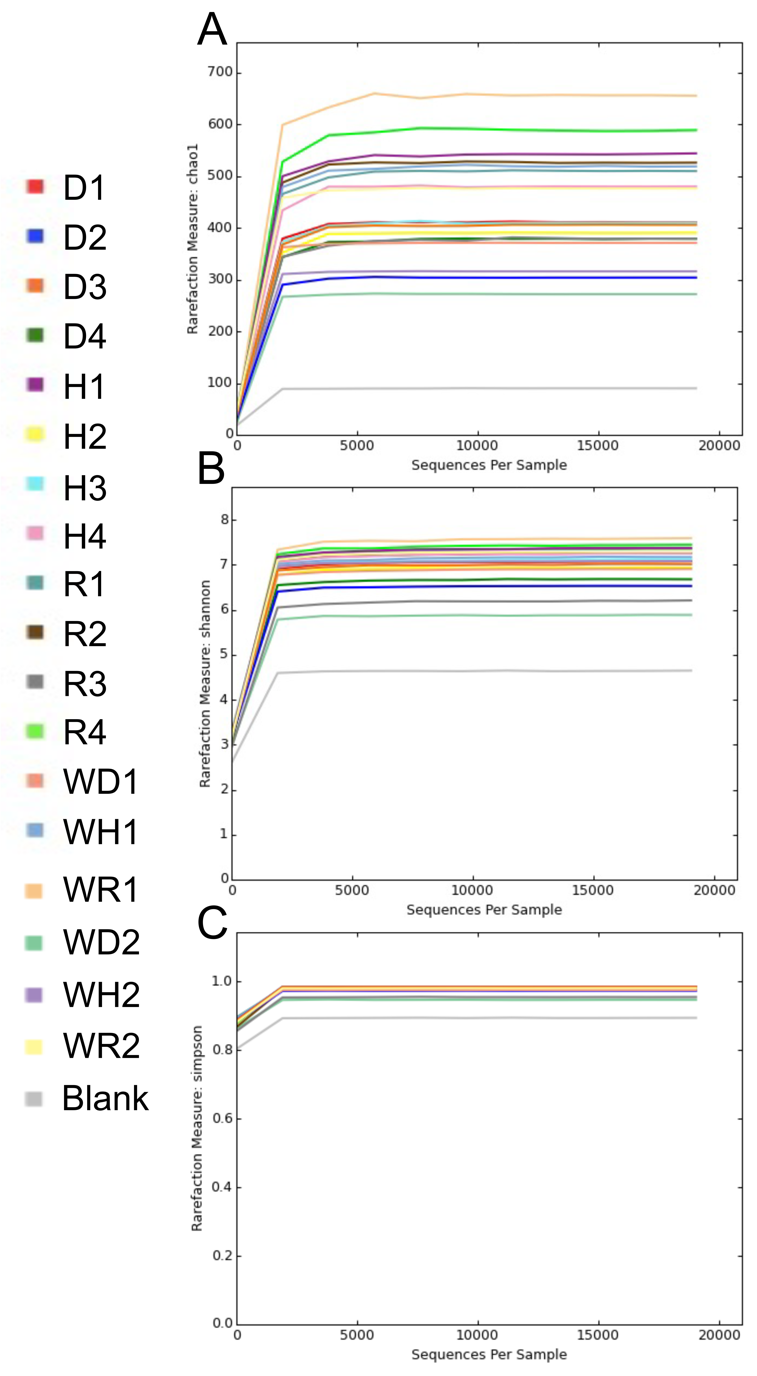


**Fig. S1.** Sufficient sampling depth is reached for each sample. Rarefaction curves for all samples analyzed in **(A)** the Chao1 index, **(B)** the Shannon index, and **(C)** the Simpson index reach a plateau, indicating that sufficient numbers of ASVs have been acquired for each sample for fair comparisons in subsequent analyses. Definitions of sample abbreviations can be found in the Methods section of the main paper.


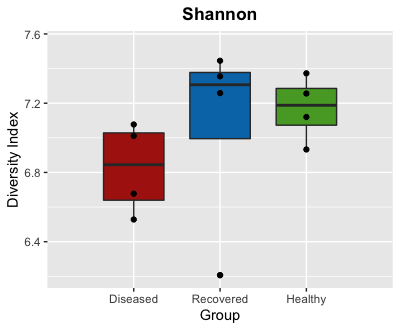


B. Shannon

A. Chao1


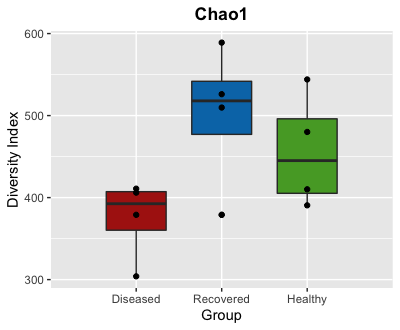

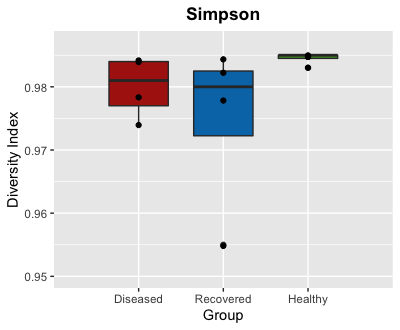


C. Simpson

Diseased

Recovered

Healthy

Diseased

Recovered

Healthy

Diseased

Recovered

Healthy

600

500

400

300

Diversity index

7.6

7.2

6.8

6.4

0.98

0.97

0.96

0.95

**Fig. S2.** Alpha diversity of the microbiomes is not different among groups. Diversity was analyzed by **(A)** Chao1, **(B)** Shannon and **(C)** Simpson indices. The Chao1 metric (Chao, 1984) examines species richness by estimating the number of ASVs in a sample based on total ASVs identified, and a greater Chao1 value indicates that a greater number of ASVs were identified. The Shannon index (Shannon, 1948) places more weight on species richness and measures the degree of uncertainty in predicting to which group an ASV belongs when selected randomly. The number for this index increases as number of species increases and as the species evenness becomes more even. The Simpson index (Simpson, 1949) measures the probability that two different selected ASVs will belong to different groups, placing more weight on species evenness than richness. The index is measured on a scale of 0-1 where 0 indicates no diversity and 1 is the highest level of diversity. The box plots show the average and quartile values for each group. There are no significant differences among the groups (ANOVA, *p* > 0.05), indicating that the alpha diversity is similar among the microbiomes of diseased, recovered and healthy groups of sea urchins.


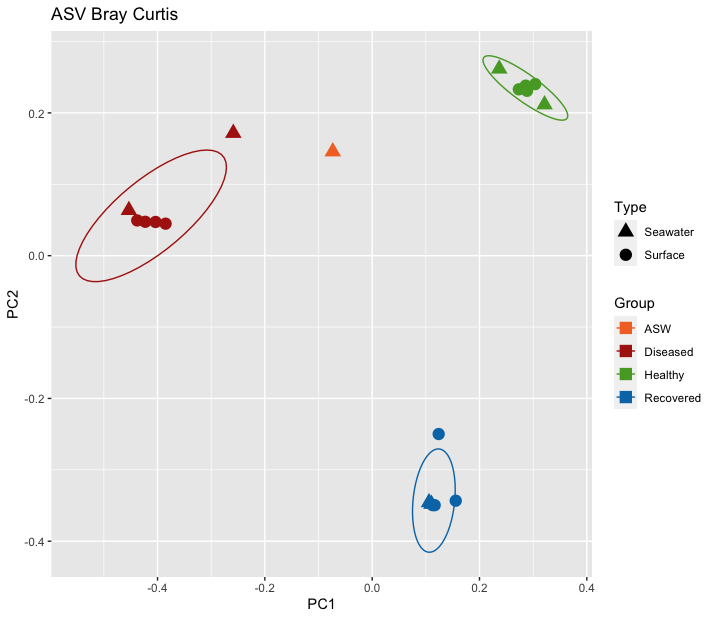


0.2

0.0

-0.2

-0.4

-0.2

0.0

0.2

0.4

PC1 (40.09%)

-0.4

PC2 (26.45%)

Seawater

Surface

ASW

Diseased

Healthy

Recovered

Bray Curtis

Sample type

Group

**Fig. S3.** The microbial compositions are different among the microbiomes of the three groups of sea urchins. See Methods section above for details. Samples within each group tend to cluster together and ellipses show the 95% confidence interval. The three groups are widely separated from one another, indicating that the microbial compositions of the three groups are significantly different (PERMANOVA, *p* < 0.05). The legend indicates Sample Type, which refers to the method of sampling. Seawater refers to samples taken from the aquarium seawater, and Surface refers to samples collected by pouring aquarium seawater over the surface of the sea urchins. The dashed box surrounding samples of the recovered group includes four data points that overlap, of which two are sea urchin surface samples and two are seawater samples.

**Fig. S4.** The most abundant phyla in the surface microbiomes are Proteobacteria and Bacteroidota. The phyla shown have an average relative abundance of > 0.1% across all samples (Table S2). The taxa are shown as the average relative abundance per sample group. “Other” encompasses all ASVs that could not be identified as a specific taxon or placed in a taxonomic group.

**Fig. S5.** The most abundant taxa are different in the microbiome samples collected from the three groups of sea urchins. Those taxa with > 2% average abundance in at least one group (Table S3) were selected to show their average relative abundances for **(A)** genus and **(B)** species for the diseased, recovered, and healthy groups. Taxa are ordered from least to most abundant in the diseased group for each taxonomic level. “Other” encompasses all ASVs that could not be identified as a specific taxon or placed in a taxonomic group.

**Fig. S6.** Taxa have different abundances in microbiomes from samples collected from diseased sea urchins compared to recovered sea urchins and from recovered sea urchins compared to healthy sea urchins. **Methods**. Those taxa with > 2% average abundance (Table S3) were selected from samples collected from **(A)** the diseased and the recovered groups of sea urchins or **(B)** the recovered and the healthy groups of sea urchins. The average relative abundances are shown for genus and species. For the diseased vs. recovered group comparison, the taxa that are most elevated in the diseased group for each taxonomic level are shown at the top and are ordered from greatest to least abundance towards the bottom. The taxa that are most elevated in the recovered group are shown on the bottom and are ordered from least to greatest abundance towards the top. For the recovered vs. healthy group comparison, the taxa that are most elevated in the healthy group at each taxonomic level are shown at the top and ordered from greatest to least abundance towards the bottom. The taxa that are most elevated in the recovered group for each taxonomic level are shown on the bottom and ordered from least to greatest abundance towards the top. The taxa labelled “Other” encompasses ASVs that could not be identified as a specific taxon or placed in a taxonomic group. **Results**. **(A)** A comparison of the taxonomic differences between the samples from the diseased group and the recovered group of sea urchins was carried out to identify differences between these microbiomes, which was based on samples that were taken at different times from sea urchins housed in the same aquarium. Results show that the genera *Colwellia*, *Erwinia*, *Leucothrix*, *Lutibacter*, and the species *Erwinia rhapontici*, *Colwellia meonggei*, *Leucothrix mucor*, and *Lutibacter agarilyticus* are elevated in abundance in the diseased group microbiomes compared to the recovered group microbiomes. These are notable taxa in the microbiomes on diseased sea urchins because they are also identified as biomarkers in the diseased group by LEfSe (Tables S4, S5). **(B)** Comparisons were also carried out for the taxa in the microbiomes of the recovered and the healthy groups of sea urchins, which were housed in different aquaria. The genera *Alteromonas*, *Cobetia*, and *Sulfurimonas* in the recovered group microbiome are both highly abundant and identified by LEfSe as biomarkers, whereas in the healthy group microbiome, *Neiella* and *Pseudoalteromonas* are identified as biomarkers and are highly abundant. The differences in bacterial abundance between the microbiomes of the three groups are in agreement with beta diversity results and illustrates the key taxa underlying the microbial differences. Overall, the pairwise comparison of the microbiomes between the diseased group vs. the recovered group, and between the recovered group vs. the healthy group show that as sea urchins transitioned from infection to recovery, their microbiomes undergo major shifts in bacterial abundance. Furthermore, sea urchins from different shipments and housed in the two different aquaria also have different microbial compositions.

5

10

5

10

15

10

5

Composition (%)

Composition (%)

Composition (%)

H1

H1

H1

ASW

WR2

WR1

R3

R4

R2

R1

D3

D4

WD1

WD2

D1

D2

WH2

WH1

H4

H3

H2

*Colwellia*

A

ASW

WR2

WR1

R3

R4

R2

R1

D3

D4

WD1

WD2

D1

D2

WH2

WH1

H4

H3

H2

*Leucothrix*

B

ASW

WR2

WR1

R3

R4

R2

R1

D3

D4

WD1

WD2

D1

D2

WH2

WH1

H4

H3

H2

*Erwinia*

C

ASW

WR2

WR1

R3

R4

R2

R1

D3

D4

WD1

WD2

D1

D2

WH2

WH1

H4

H3

H2

H1

*Desulfotalea*

Composition (%)

7.5

5

2.5

D

ASW

WR2

WR1

R3

R4

R2

R1

D3

D4

WD1

WD2

D1

D2

WH2

WH1

H4

H3

H2

H1

*Sulfurimonas*

Composition (%)

5

10

15

F

ASW

WR2

WR1

R3

R4

R2

R1

D3

D4

WD1

WD2

D1

D2

WH2

WH1

H4

H3

H2

H1

*Spirochaeta*

0.5

1

1.5

2

Composition (%)

E

I

G

ASW

WR2

WR1

R3

R4

R2

R1

D3

D4

WD1

WD2

D1

D2

WH2

WH1

H4

H3

H2

H1

Composition (%)

2.5

5

7.5

10

ASW

WR2

WR1

R3

R4

R2

R1

D3

D4

WD1

WD2

D1

D2

WH2

WH1

H4

H3

H2

H1

2

3

1

Composition(%)

ASW

WR2

WR1

R3

R4

R2

R1

D3

D4

WD1

WD2

D1

D2

WH2

WH1

H4

H3

H2

H1

*Arcobacter*

Composition(%)

1

2

3

4

H

*Rubritalea*

*Vibrio*

Species

Species

Species

Species

Species

Species

**Fig. S7.** Some taxa show differences in the microbiomes of the diseased compared to the recovered groups, plus differences based on different shipments of sea urchins housed in two aquaria. **(A-C)** Genera *Colwellia*, *Leucothrix* and *Erwinia* show elevated abundance in the microbiomes from the diseased group compared to the microbiomes from recovered group, and are not influenced by shipment and housing differences. These taxa may be associated with the transition from disease to recovery, particularly because the abundances of these taxa are similar for samples from the recovered and healthy groups. **(D, E)** Genera that show reduced abundance in the microbiome of the diseased group compared to the microbiome of the recovered group. *Desulfotalea* and *Spirochaeta* are associated with the transition to recovery but do not differ between the recovered and healthy groups, indicating that the changes to the abundance of these taxa are correlated with recovery from BSUD. **(F)** The *Sulfurimonas* genus demonstrates differences in abundance in the microbiome of the diseased group compared to the recovered group. It also shows differences in the microbiomes of recovered and healthy sea urchins in the two aquaria, indicating that the changes in abundance are due to a combination of recovery from disease and differences between sea urchin shipments. **(G, H)** Genera *Rubritalea* and *Arcobacter* do not show differences in abundance in the microbiome of the diseased group compared to the recovered group but do show differences based on the different shipments of sea urchins housed in different aquaria. The abundances of these taxa correlate with different shipments and not the transition to recovery. **(I)** The *Vibrio* genus does not show differences with respect to disease and recovery or to differences based on shipments and housing, but instead is abundant across all groups. This suggests that it may not be involved in the progression of BSUD. All ASVs for each genus are included, and each bar indicates their composition as a percent of total microbial composition for each sea urchin in a group plus the corresponding seawater samples. All samples are shown, including the ASW control, which is prepared from freshly mixed Omega seawater. See the Materials and Methods section in the main paper for definitions of sample abbreviations.

ASW

WR2

WR1

R3

R4

R2

R1

D3

D4

WD1

WD2

D1

D2

WH2

WH1

H4

H3

H2

H1

*Algibacter*

Composition (%)

1

2

3

4

5

A

ASW

WR2

WR1

R3

R4

R2

R1

D3

D4

WD1

WD2

D1

D2

WH2

WH1

H4

H3

H2

H1

*Fluviicola*

Composition (%)

4

3

2

1

C

ASW

WR2

WR1

R3

R4

R2

R1

D3

D4

WD1

WD2

D1

D2

WH2

WH1

H4

H3

H2

H1

*Lutibacter*

Composition (%)

6

4

2

B

*Polaribacter*

*Octadecabacter*

E

ASW

WR2

WR1

R3

R4

R2

R1

D3

D4

WD1

WD2

D1

D2

WH2

WH1

H4

H3

H2

H1

Composition (%)

0.5

1

D

ASW

WR2

WR1

R3

R4

R2

R1

D3

D4

WD1

WD2

D1

D2

WH2

WH1

H4

H3

H2

H1

Composition (%)

1

0.5

F

ASW

WR2

WR1

R3

R4

R2

R1

D3

D4

WD1

WD2

D1

D2

WH2

WH1

H4

H3

H2

H1

Composition (%)

0.3

0.6

0.9

Composition (%)

*Blastopirellula*

Species

Species

Species

Species

**Fig. S8.** Some genera show elevated abundance in the microbiomes from the diseased group relative to the microbiome from the recovered group. Selected taxa (> 1% of the total taxa of a group) are shown that have higher abundances in the microbiome of the diseased group compared to the recovered group. **(A)** The *Algibacter* genus shows an elevated abundance in the microbiome of the diseased group, and is not influenced by differences based on different shipments of sea urchins housed in different aquaria. **(B-F)** Examples of genera that have elevated abundances in the microbiome of the diseased group compared to the recovered group also show differences in abundances for the recovered group compared to the healthy group in the two aquaria. All ASVs for each genus are included, and each bar indicates their composition as a percent of total microbial composition for each sea urchin in a group plus the corresponding seawater samples. All samples are shown for each panel, including the ASW control, which is prepared from freshly mixed Omega seawater. See the Materials and Methods section in the main paper for definitions of sample abbreviations.

**Supplementary Tables**

**Sequencing Results**. Sequencing of all gDNA samples resulted in a total of 2,443,194 raw sequence reads. All sequencing errors and chimeric sequences were removed and sequence trimming was performed, resulting in a total of 8,027 unique sequences. After taxonomic assignment, 1,982 ASVs were identified across all samples. Within these ASVs, 25 phyla, 47 classes, 92 orders, 170 families, 388 genera, and 1,057 species were identified.

**Table S1.** ASVs^1^ are identified in each sample

| **Sample ID^2^** | **Raw seqs** | **trimmed** | **dada2_ inferred** | **Chimeric seqs** | **Chimera free seqs** | **Unique seqs** | **Seqs (after size filtration)** | **Final unique seqs** |
| --- | --- | --- | --- | --- | --- | --- | --- | --- |
| D1 | 124082 | 122272 | 56056 | 7486 | 48570 | 674 | 47091 | 411 |
| D2 | 118654 | 117102 | 50173 | 14921 | 35252 | 559 | 33922 | 304 |
| D3 | 150108 | 147632 | 67066 | 12751 | 54315 | 644 | 52967 | 406 |
| D4 | 146880 | 144634 | 67789 | 9385 | 58404 | 626 | 57112 | 379 |
| WD1 | 103490 | 101584 | 42134 | 10513 | 31621 | 701 | 29785 | 371 |
| WD2 | 103950 | 102592 | 45327 | 12539 | 32788 | 532 | 31496 | 272 |
| R1 | 122538 | 120440 | 55838 | 5965 | 49873 | 850 | 47810 | 510 |
| R2 | 120816 | 119004 | 55281 | 5858 | 49423 | 891 | 47277 | 526 |
| R3 | 149270 | 147042 | 70090 | 6058 | 64032 | 642 | 62490 | 379 |
| R4 | 158712 | 156542 | 72507 | 8175 | 64332 | 962 | 62106 | 589 |
| WR1 | 148810 | 146192 | 67561 | 7744 | 59817 | 1033 | 57582 | 656 |
| WR2 | 106176 | 104318 | 46056 | 8452 | 37604 | 848 | 35536 | 476 |
| H1 | 141568 | 139300 | 63584 | 6315 | 57269 | 906 | 55185 | 543 |
| H2 | 152366 | 150254 | 69510 | 11287 | 58223 | 631 | 56825 | 390 |
| H3 | 127584 | 125410 | 57288 | 7029 | 50259 | 673 | 48785 | 410 |
| H4 | 129052 | 126848 | 57726 | 6132 | 51594 | 798 | 49709 | 480 |
| WH1 | 139740 | 137488 | 63203 | 6994 | 56209 | 827 | 54436 | 519 |
| WH2 | 62948 | 62028 | 25430 | 4878 | 20552 | 599 | 19057 | 316 |
| ASW | 136450 | 133846 | 65705 | 4105 | 61600 | 99 | 60584 | 90 |
| Total | 2443194 | 2404528 | 1098324 | 156587 | 941737 | 13495 | 909755 | 8027 |

^1^Amplicon sequence variants

^2^Abbreviations are defined in the materials and methods section in the main paper.

**Table S2.** Phyla of highest abundance in surface microbiome samples

| **Taxa^1^** | **Diseased^2^** | **Recovered** | **Healthy** |
| --- | --- | --- | --- |
| Proteobacteria | 64.444 | 64.90 | 61.14 |
| Other | 10.90 | 14.43 | 19.30 |
| Bacteroidetes | 17.15 | 5.25 | 11.79 |
| Verrucomicrobia | 3.19 | 3.37 | 0.84 |
| Lentisphaerae | 0.41 | 4.22 | 1.59 |
| Planctomycetes | 0.18 | 2.84 | 1.59 |
| Spirochaetae | 0.01 | 1.88 | 1.04 |
| Firmicutes | 0.39 | 0.93 | 1.52 |
| Actinobacteria | 0.46 | 0.27 | 0.33 |
| Thaumarchaeota | 0.02 | 0.55 | 0.27 |
| Gracilibacteria | 0.06 | 0.56 | 0.10 |
| Chloroflexi | 0.42 | 0.10 | 0.06 |
| Cyanobacteria | 0.17 | 0.10 | 0.15 |
| Saccharibacteria | 0.20 | 0.17 | 0.02 |
| Chlamydiae | 0.13 | 0.11 | 0.07 |

^1^Phyla were selected that have an relative abundance of >0.1% for at least one sample

^2^Abundance is indicated as the average percentage (%) of four sea urchin samples of the total taxa identified within each group.

**Table S3.** Bacterial taxa of > 2% relative abundance^1^ in each sample group

| **Taxon level** | **Sample Group** | | | | | |
| --- | --- | --- | --- | --- | --- | --- |
|  | **Diseased** | **%^1^** | **Recovered** | **%** | **Healthy** | **%** |
| Genus | Other | 10.90 | *Psychromonas* | 15.97 | Other | 19.30 |
|  | *Colwellia* | 10.44 | Other | 14.23 | *Psychromonas* | 13.13 |
|  | *Psychromonas* | 8.83 | *Alteromonas* | 8.83 | *Vibrio* | 5.57 |
|  | *Erwinia* | 8.81 | *Sulfurimonas* | 5.00 | Unknown | 3.50 |
|  | *Leucothrix* | 6.92 | *Desulfotalea* | 4.33 | *Desulfotalea* | 3.27 |
|  | Unknown | 4.91 | Unknown | 3.07 | Unknown | 2.98 |
|  | *Vibrio* | 4.85 | *Vibrio* | 2.52 | *Colwellia* | 2.91 |
|  | *Lutibacter* | 3.33 | *Cobetia* | 2.36 | *Parvularcula* | 2.87 |
|  |  |  |  |  | *Pseudoalteromonas* | 2.55 |
|  |  |  |  |  | *Neiella* | 2.50 |
|  |  |  |  |  |  |  |
| Species | Other | 10.90 | Other | 14.43 | Other | 19.30 |
|  | *Erwinia rhapontici* | 8.81 | *Psychromonas profunda* | 12.82 | *Psychromonas profunda* | 9.10 |
|  | *Leucothrix mucor* | 5.47 | *Alteromonas sp* | 6.30 | *Desulfotalea sp52096* | 3.27 |
|  | Genus unknown *sp18586* | 4.83 | *Sulfurimonas sp55735* | 4.98 | Genus unknown  *sp11768-11813* | 2.96 |
|  | *Psychromonas profunda* | 4.83 | *Desulfotalea sp52097* | 3.80 | *Psychromonas heitensis kaikoae* | 2.93 |
|  | *Lutibacter agarilyticus* | 3.24 | *Psychromonas kaikoae* | 2.28 | *Parvularcula sp43129* | 2.70 |
|  | *Colwellia meonggei sp56501* | 2.86 | Genus unknown *sp57249* | 2.25 | Genus unknown *sp60188* | 2.68 |
|  | *Colwellia* *psychrerythraea* | 2.83 |  |  | *Neiella* *sp56428* | 2.50 |
|  | *Psychromonas* *arctica* | 2.54 |  |  | *Pseudoalteromonas* *sp* | 2.08 |
|  | *Colwellia* *aestuarii* | 2.27 |  |  |  |  |

^1^Abundance is indicated as the average percentage (%) of four sea urchin samples of the total taxa identified within each group.

**Table S4**. Biomarker taxa of significantly different abundances and large effect size

| **Group** | **Species** | **LDA^1^ score** | ***p* value^2^** |
| --- | --- | --- | --- |
| Diseased | *Colwellia meonggei sp56501* | 4.4652 | 0.0105 |
|  | *Lutibacter agarilyticus* | 4.4461 | 0.0119 |
|  | *Fluviicola sp16216* | 4.3087 | 0.0061 |
|  | *Leucothrix mucor sp64100* | 4.1732 | 0.0073 |
|  | *Colwellia meonggei sp56501-sp56505* | 4.0243 | 0.0210 |
|  | *Blastopirellula sp41404-sp41424* | 3.7883 | 0.0244 |
|  | *Arcobacter sp55236* | 3.7812 | 0.0231 |
|  | *Erwinia rhapontici* | 3.7566 | 0.0159 |
|  | Unknown genus, Family *Oceanospirillaceae* | 3.2464 | 0.0092 |
|  | Unknown genus, Family *Saprospiraceae* | 2.9709 | 0.0285 |
| Recovered | *Alteromonas sp* | 4.7249 | 0.0183 |
|  | *Francisella persica tularensis* | 4.1164 | 0.0061 |
|  | *Rubritalea sp69317-sp69326* | 4.0694 | 0.0231 |
|  | Unknown genus, Family *Cellvibrionaceae* | 3.7218 | 0.0061 |
|  | *Cobetia litoralis marina* | 3.7163 | 0.0163 |
|  | *Kordiimonas sp43012* | 3.2984 | 0.0158 |
|  | *Vibrio panuliri* | 3.2208 | 0.0106 |
|  | *Arenicella xantha* | 3.1722 | 0.0158 |
|  | Unknown genus, Family *Nannocystaceae* | 3.1601 | 0.0158 |
|  | *Roseibacillus ponti* | 3.1160 | 0.0397 |
|  | *Nitrosoarchaeum koreensis* | 3.0930 | 0.0158 |
|  | *Phycisphaera sp40965* | 3.0907 | 0.0321 |
|  | *Rhodopirellula sp41964-sp42046* | 3.0882 | 0.0227 |
|  | *Altererythrobacter sp47504* | 3.0686 | 0.0201 |
|  | *Coxiella sp60512* | 3.0519 | 0.0105 |
|  | *Coxiella sp60417-sp60493-sp60525* | 2.9418 | 0.0469 |
|  | *Thiohalophilus sp60165* | 2.9317 | 0.0426 |
|  | *Roseibium aquae* | 2.8009 | 0.0441 |
| Healthy | *Psychromonas heitensis kaikoae* | 4.5119 | 0.0231 |
|  | *Prolixibacter sp* | 4.0116 | 0.0488 |
|  | *Oceaniovalibus sp44845* | 3.8855 | 0.0154 |
|  | *Desulfopila Desulfotalea sp52047-sp52096* | 3.8490 | 0.0092 |
|  | *Sulfurimonas sp55715-sp55735* | 3.8380 | 0.0092 |
|  | *Neiella* *sp* | 3.8231 | 0.0090 |
|  | *Loktanella cinnabarina hongkongensis* | 3.8146 | 0.0105 |
|  | *Spirochaeta litoralis* | 3.7597 | 0.0061 |
|  | *Litoribacillus sp61538-sp61539* | 3.7179 | 0.0061 |
|  | *Marinobacter salarius* | 3.6288 | 0.0073 |
|  | *Vibrio litoralis* | 3.6156 | 0.0123 |
|  | *Marinobacter salarius similis* | 3.5558 | 0.0090 |
|  | *Halomonas denitrificans saccharevitans* | 3.4472 | 0.0426 |
|  | *Halomonas sp* | 3.4304 | 0.0285 |
|  | *Saccharicrinis sp12936* | 3.3791 | 0.0321 |
|  | *Vibrio atypicus panuliri* | 3.3075 | 0.0106 |
|  | *Shewanella olleyana* | 3.2845 | 0.0123 |
|  | *Draconibacterium sp14871* | 3.2565 | 0.0090 |
|  | *Marinosulfomonas methylotropha* | 3.1577 | 0.0092 |
|  | *Planctomyces sp* | 3.1201 | 0.0092 |
|  | *Halomonas alkaliantarctica boliviensis* | 3.1154 | 0.0092 |
|  | *Mycobacterium hippocampi* | 3.0462 | 0.0157 |
|  | *Nitrospira sp39765* | 2.9982 | 0.0344 |

^1^Linear Discriminant Analysis

^2^*p* value from the non-parametric factorial Kruskal-Wallis (KW) sum-rank test indicates that the taxa listed are significantly differentially abundant for each group.

**Table S5.** Biomarker genera of significantly different abundances and large effect size

| **Group** | **Genus** | **LDA score^1^** | ***p* value^2^** |
| --- | --- | --- | --- |
| Diseased | *Colwellia* | 4.6466 | 0.0209 |
|  | *Lutibacter* | 4.4476 | 0.0183 |
|  | *Fluviicola* | 4.2969 | 0.0061 |
|  | *Leucothrix* | 4.1674 | 0.0073 |
|  | *Octadecabacter* | 4.0431 | 0.0146 |
|  | *Arcobacter* | 3.7800 | 0.0231 |
|  | *Blastopirellula* | 3.7794 | 0.0244 |
|  | *Erwinia* | 3.7562 | 0.0159 |
|  | Unknown, Family *Oceanospirillaceae* | 3.3865 | 0.0092 |
|  | *Polaribacter* | 3.2634 | 0.0092 |
|  | Unknown, Family *Bdellovibrionaceae* | 3.0068 | 0.0089 |
|  | Unknown, Family *Saprospiraceae* | 2.9545 | 0.0285 |
| Recovered | *Alteromonas* | 4.7061 | 0.0209 |
|  | *Sulfurimonas* | 4.6135 | 0.0182 |
|  | *Rubritalea* | 4.1317 | 0.0231 |
|  | *Francisella* | 4.0992 | 0.0236 |
|  | *Cobetia* | 3.8343 | 0.0163 |
|  | Unknown, Family *Cellvibrionaceae* | 3.7245 | 0.0061 |
|  | *Kordiimonas* | 3.2725 | 0.0158 |
|  | *Altererythrobacter* | 3.2152 | 0.0158 |
|  | *Coxiella* | 3.1917 | 0.0183 |
|  | *Arenicella* | 3.1851 | 0.0158 |
|  | *Desulfovibrio* | 3.1556 | 0.0105 |
|  | Unknown, Family *Nannocystaceae* | 3.1431 | 0.0158 |
|  | *Nitrosoarchaeum* | 3.0996 | 0.0158 |
|  | *Roseibacillus* | 3.0629 | 0.0397 |
|  | *Winogradskyella* | 3.0517 | 0.0360 |
|  | *Thiohalophilus* | 2.8958 | 0.0426 |
|  | *Roseibium* | 2.7997 | 0.0441 |
| Healthy | *Psychromonas* | 4.4953 | 0.0097 |
|  | *Shewanella* | 4.1671 | 0.0072 |
|  | *Prolixibacter* | 4.0340 | 0.0488 |
|  | *Desulfopila Desulfotalea* | 3.9085 | 0.0092 |
|  | *Oceaniovalibus* | 3.8939 | 0.0154 |
|  | *Marinobacter* | 3.8760 | 0.0244 |
|  | *Loktanella* | 3.8399 | 0.0061 |
|  | *Neiella* | 3.8154 | 0.0089 |
|  | *Spirochaeta* | 3.7562 | 0.0061 |
|  | *Litoribacillus* | 3.6985 | 0.0061 |
|  | *Marinosulfomonas* | 3.5820 | 0.0092 |
|  | *Pseudoalteromonas* | 3.4575 | 0.0146 |
|  | *Saccharicrinis* | 3.3842 | 0.0321 |
|  | *Sulfitobacter* | 3.3788 | 0.0173 |
|  | *Draconibacterium* | 3.2783 | 0.0089 |
|  | *Gilvibacter* | 3.1898 | 0.0092 |
|  | Unknown, Family *Sandaracinaceae* | 3.1648 | 0.0231 |
|  | *Psychroflexus* | 3.0885 | 0.0148 |
|  | *Mycobacterium* | 3.0719 | 0.0157 |
|  | *Planctomyces* | 3.0488 | 0.0148 |
|  | *Nitrospira* | 2.9978 | 0.0343 |

^1^Linear discriminant analysis

^2^*p* value from the non-parametric factorial Kruskal-Wallis (KW) sum-rank test indicates that the taxa listed are significantly differentially abundant in the group of interest.

**References**

Benjamini, Y., Hochberg, Y., 1995. Controlling the false discovery rate: a practical and powerful approach to multiple testing. J. R. Stat. Soc: Series B (Methodological). 57:1, 289–300. doi: 10.1111/j.2517-6161.1995.tb02031.x

Callahan, B.J., McMurdie, P.J., Rosen, M.J., Han, A.W., Johnson, A.J.A., Holmes, S.P., 2016. DADA2: High-resolution sample inference from Illumina amplicon data. Nat. Methods. 13:7, 581–583. doi: 10.1038/nmeth.3869

Caporaso, J.G., Kuczynski, J., Stombaugh, J., Bittinger, K., Bushman, F.D., Costello, E.K., Fierer, N., Peña, A.G., Goodrich, J.K., Gordon, J.I., Huttley, G.A., Kelley, S.T., Knights, D., Koenig, J.E., Ley, R.E., Lozupone, C.A., McDonald, D., Muegge, B.D., Pirrung, M., Reeder, J., Sevinsky, J.R., Turnbaugh, P.J., Walters, W.A., Widmann, J., Yatsunenko, T., Zaneveld, J., Knight, R., 2010. QIIME allows analysis of high-throughput community sequencing data. Nat. Methods. 7:5, 335–336. doi: 10.1038/nmeth.f.303

Chao, A., 1984. Nonparametric estimation of the number of classes in a population. Scand. J. Stat. 11:4, 265–270. doi: 10.2307/4615964

Oksanen, J., Guillaume Blanchet, F., Kindt, R., Legendre, P., Minchin, P., O'Hara, B. Simpson, G., Solymos, P., Stevens, H., Wagner, H., 2015. Vegan: Community Ecology Package. R Package Version 2.2-1. 2. 1-2.

Segata, N., Izard, J., Waldron, L., Gevers, D., Miropolsky, L., Garrett, W.S., Huttenhower, C., 2011. Metagenomic biomarker discovery and explanation. Genome Biol. 12:6, R60. doi: 10.1186/gb-2011-12-6-r60

Shannon, C.E., 1948. A mathematical theory of communication. Bell Syst. Tech. J. 27:3, 379-423. doi: 10.1002/j.1538-7305.1948.tb01338.x

Simpson, E.H., 1949. Measurement of diversity. Nature. 163:4148, 688. doi: 10.1038/163688a0
