# Supplementary material for: Bald sea urchin disease shifts the surface microbiome on purple sea urchins in an aquarium
Source: Pathog Dis. 2023 Sep 15;81:ftad025. doi: 10.1093/femspd/ftad025 (PMC10550250; doi:10.1093/femspd/ftad025)
Supplement: ftad025_Supplemental_Files [file ftad025_supplemental_files.zip › Shaw etal Supplementary Data File 1.docx]

Supplementary Data File 1

**Bald sea urchin disease shifts the surface microbiome on purple sea urchins in an aquarium**

Chloe G. Shaw, Christina Pavloudi, Megan A. Barela Hudgell, Ryley S. Crow, Jimmy H. Saw, R. Alexander Pyron, L. Courtney Smith

**Method modification for collecting bacterial gDNA**

The method for DNA isolation as reported by Turner et al. (2014) was followed closely with a few modifications for isolation from microbes. Rather than storing the filter membranes after sample collection with CTAB in the freezer, the filters were stored in the freezer without CTAB. For processing, filters were brought to room temperature, placed in a plastic petri dish (10 cm diameter), and 1 ml of CTAB rather than 700 𝜇l was added to fully cover the filter surface. The dish with the filter and CTAB was placed on a hot plate at 65^o^C for 10 min with agitation. Turner et al. (2014) dissolved the filter during the cell lysis step, however, we used a cell scraper to remove the material from the filter and transferred the material in CTAB to a separate tube before cell lysis. This modification was performed to exclude the filter remnants from the DNA isolation protocol. Finally, DNA precipitation was carried out at -80^o^C for 15 minutes rather than -20^o^C for 1 hour.

To establish and verify the modifications to the method, gDNA isolation and PCR amplification of the 16S rRNA gene were optimized using *E. coli*. An overnight culture in LB was used undiluted, diluted 10X, 100X, and 1000X with deionized water (DI) and then the bacterial dilution samples were added to 200 ml DI water to enable filtering onto a 0.2 µm filter. This dilution series was done to facilitate collecting different numbers of bacteria using the vacuum filtration method as described in the main paper. The gDNA was evaluated by PCR to test whether it would support amplification of the 16S rRNA gene (see Methods in the main paper). Results showed that amplicons of the expected size could be discerned from all samples including that diluted 1000X and using 0.02 ng of gDNA template (see Fig.). This demonstrated that the approach could be used to isolate gDNA from potentially limited numbers of bacteria collected from the surface microbiome on sea urchins and from seawater from the two aquaria.

Culture dilution





1.5

A B C D E F G H I J

UD

10X

100X

1000X

**Fig. Maximum dilution of an *E. coli* culture supports gDNA isolation and 16S rRNA gene amplification.** A range of dilutions of an overnight culture of *E. coli* (indicated; UD, undiluted) are used to evaluate the method for isolating gDNA from bacteria collected on filters. The amount of gDNA used as template in PCR is 2.0 ng (lanes A, D,), 0.2 ng (lanes B, E, G, I), and 0.02 ng (lanes C, F, H, J). The expected 16S rRNA gene amplicon size is 1.5 kb. The presence of bands at all dilutions indicates that low numbers of bacterial cells are sufficient for gDNA isolation and PCR amplification.

**Reference**

Turner, C.R., Miller, D.J., Coyne, K.J., Corush, J., 2014. Improved methods for capture, extraction, and quantitative assay of environmental DNA from Asian bigheaded carp (*Hypophthalmichthys spp.).* PLoS ONE. 9:12, e114329. doi: 10.1371/journal.pone.0114329
